# Microbiota-derived butyrate inhibits cDC development via HDAC inhibition, diminishing their ability to prime T cells

**DOI:** 10.1016/j.mucimm.2024.08.003

**Published:** 2024-12

**Authors:** Anna Andrusaite, Jennifer Lewis, Annika Frede, Andrew Farthing, Verena Kästele, Jennifer Montgomery, Allan Mowat, Elizabeth Mann, Simon Milling

**Affiliations:** a School of Infection and Immunity, University of Glasgow, UK; b Lydia Becker Institute of Immunology and Inflammation, University of Manchester, UK

## Abstract

Conventional dendritic cells (cDC) are central to maintaining the balance between protective immune responses and tolerance to harmless antigens, especially in the intestine. Short chain fatty acids (SCFAs) such as butyrate play critical roles in regulating intestinal immunity, but the underlying mechanisms remain unclear. Here we demonstrate that microbiota-derived butyrate alters intestinal cDC populations *in vivo* resulting in decreased numbers of the cDC2 lineage. By establishing a novel *in vitro* culture model, we show that butyrate has a direct and selective ability to repress the development of cDC2 from cDC precursors, an effect that is independent of G-protein coupled receptors (GPCRs) and is due to inhibition of histone deacetylase 3. Finally, cDC derived from pre-cDC in the presence of butyrate *in vitro* express lower levels of costimulatory molecules and have a decreased ability to prime naïve T cells. Together, our data show that butyrate affects the developmental trajectory of cDC, selectively repressing the cDC2 lineage and reducing their ability to stimulate T cells. These properties may help explain the ability of butyrate to maintain homeostasis in the intestine.

## Graphical abstract

**Figure:**
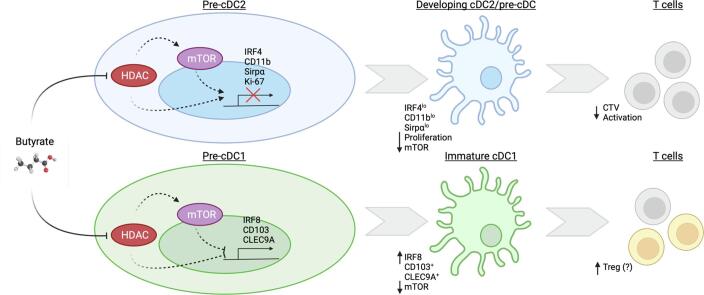

## Introduction

Conventional dendritic cells (cDC) in the intestinal mucosa contribute to local homeostasis by secreting cytokines, sampling luminal contents and migrating to the draining mesenteric lymph node (MLN), where they present antigen to naive T cells. Intestinal cDC arrive in the tissue as bone marrow (BM) derived precursors (pre-cDC) and complete their maturation and activation in the intestinal lamina propria (LP).^1^, ^2^ As in other tissues, the local environment determines how intestinal cDC behave, with tolerance to food and microbiota-derived antigens occurring under steady state conditions, whereas inflammation or infection stimulate cDC to drive effector T cell development. The factors controlling this plasticity of tissue specific cDC are not yet fully understood and here we have explored how microbiota-derived short chain fatty acids (SCFAs), might contribute to the development and activity of cDC in the intestine.

We have previously shown that manipulation of SCFA abundance in the intestine can have an effect on myeloid cell composition and activity.^3^ SCFAs can also increase the number of circulating monocytes and polarise macrophages towards an alternatively activated state.^4^, ^5^, ^6^ As for cDC, the cDC compartment is overall altered GF animals^7^ as well as upon microbiota depletion using antibiotics.^8^ Furthermore SCFA modulation in the gut also affects cDC composition in colon-draining lymph nodes.^9^, ^10^ In parallel, SCFAs can alter the abundance of effector T cells in the intestine,^11^ and the frequency of inducible regulatory T cells (Tregs) correlates with the luminal concentrations of SCFAs.^12^ Furthermore, the absence of Tregs in germ free (GF) mice can be restored by administration of SCFAs.^13^ Although modulation of intestinal SCFA levels alters the composition of cDC populations in colon-draining lymph nodes,^9^, ^10^ less is known about the direct effects of SCFAs on cDC. Furthermore, their effects on cDC at their primary site of development, the colonic LP, have not been studied. In the above described studies, butyrate was shown to have a particulary strong influence on immune functions, due to its dual ability to both signal through the G-protein coupled receptors (GPCRs) and act as a histone deacetylase inhibitor (HDACi).

Here we hypothesised that SCFAs would have direct effects on cDC and on their ability to prime T cell responses. We first investigated how butyrate, the SCFA with the most potent effects on the immune response,^5^, ^14^, ^15^ can affect local intestinal LP cDC populations *in vivo.* We then utilised a novel approach for generating cDC *in vitro* to explore further the effects of butyrate on cDC development, phenotype and function. To test which signalling mechanism underlie the changes observed we used a combination of GPCR knock out animals, GPCR inhibitors, and HDAC inhibitors. Finally, we investigated how butyrate affects cDC functions by co-culturing cDC with naïve T cells. Overall, we demonstrate effects of butyrate on cDCs, which provide a mechanistic explanation for the previously observed corelations between SCFA abundance and anti-inflammatory immune responses in the intestine.

## Results

### Effects of butyrate administration on the composition of intestinal dendritic cell populations

To understand how microbiota and SCFA might influence the intestinal cDC niche, we first analysed a publicly available single cell transcriptome data set comparing colonic cDC from GF and specified pathogen free (SPF) animals (Figure S1). In our re-analysis cDC were identified using the well-defined genes *Zbtb46*, *Flt3l*, *Itgax* (coding CD11c), *Cd24a* and *Kit*,^16^ revealing 10 cDC clusters with differentially expressed genes (Fig. S1a). Of these, clusters 1,3,4,10 were annotated as cDC1 based on their expression of *Xcr1*, *Clec9a* and *Irf8*, whereas clusters 0,2,5,6,7,8,9 were identified as cDC2 based on their expression of *Irf4* and *Sirpα* and the absence of cDC1 lineage markers. Further analysis showed that the GF colon had an increased proportion of total cDC2 compared with SPF colon, with a corresponding decrease in total cDC1 (Fig. S1b,c). All 4 cDC1 clusters followed the same pattern of decrease in GF mice (Fig. S1d,e). Six of the seven cDC2 clusters were increased in GF vs SPF colon. Cluster 9 did not follow the pattern of the other cDC2 clusters. It was defined by genes such as *Ccnd2, Nfil3* and *Bcl2a1a* (Fig. S1a) that regulate cell cycle,^17^ cDC development^18^ and survival^19^ and may therefore represent a cDC precursor population. Together, this data show that in the presence of microbiota the development and composition of colon cDC may be altered.

**Fig. 1:**
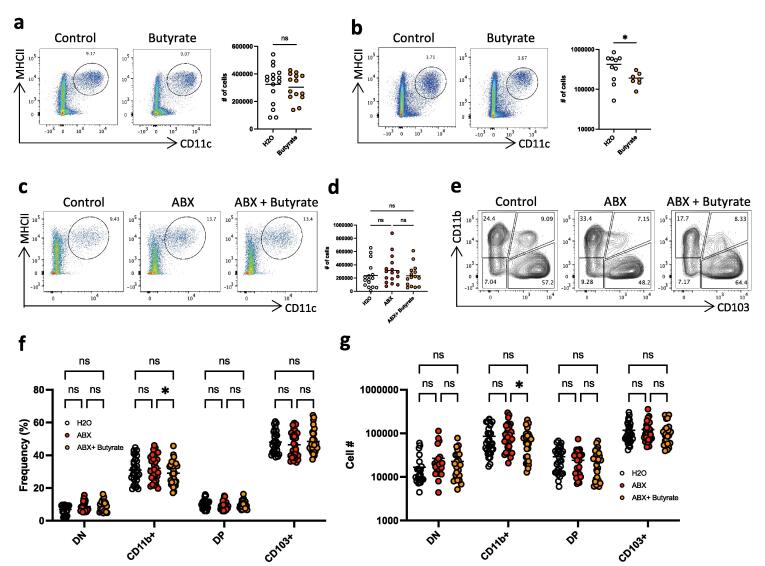
Relationship between intestinal dendritic cell populations and abundance of SCFAs *in vivo*. A) Representative FACS plots (left panels) and numbers (right panel) of colonic and B) small intestinal LP cDC in control mice or in mice supplemented with 200 mM butyrate via the drinking water. cDC were identified as live, single, CD45+, CD3-, B220-, CD64-, MHCII+and CD11c + . Data shown are pooled from 2 to 4 independent experiments (n = 3–4 each) C) Representative FACS plots and D) numbers of colonic LP cDC in control mice, in mice treated with antibiotic cocktail (ABX) and in mice treated with antibiotics and supplemented with 200 mM butyrate (ABX+Butyrate). The data shown are pooled from 4 independent experiments (n = 3–5 each) E) Representative FACS plots of colonic LP cDC subsets based on their CD11b and CD103 expression in control mice, in mice treated with antibiotic cocktail (ABX) and in mice treated with antibiotics and supplemented with 200 mM butyrate (ABX+Butyrate). F) Frequencies amongst total cDC and G) absolute numbers of colonic LP cDC subsets in control mice, in mice treated with antibiotic cocktail (ABX) and in mice treated with antibiotics and supplemented with 200 mM butyrate (ABX+Butyrate). Cells pre-gated on live, single, CD45^+^, B220^-^, CD3^-^, CD64^-^, CD11c^+^, MHC^+^ cells. DN=CD11b^-^CD103^-^ double negative, single CD11b^+^ = CD11b^+^CD103^-^, DP=CD11b^+^CD103^+^ double positive and single CD11b^-^CD103^+^ (CD103^+^). The data shown are from 7 independent experiments (n = 3–5 each). *p < 0.05, as assessed by Student’s *t*-test (A, B), one-way ANOVA (D) or two-way ANOVA with Šídák’s post-test correction for multiple comparisons (F, G). ns = not significant.

To explore the possibility that SCFA might be one factor involved in the above observed processes, we first examined the effects of increasing the local levels of the SCFA butyrate *in vivo* by adding it to the drinking water of steady state C57/Bl6 mice for 7 days.^3^ Lamina propria cells were isolated from colon and small intestine (SI), and cDC were identified as live, CD45^+^ B220^-^ CD3^-^ CD64^-^ CD11c^+^ MHC II^+^^20^, ^21^ (Figure S2). Butyrate administration had no effect on the total number of cDC in the colonic LP (Fig. 1a), but there was a significant decrease in the numbers of cDC in the small intestinal LP (Fig. 1b). As the standard chow diet contains high levels of fibre, we hypothesised that SCFA concentrations would already be maximal in the microbiota-rich colon but not in the SI and that this could have masked any effect of exogenous butyrate supplementation.

To examine whether this might explain the difference between colon and SI, we depleted the microbiota by administering a mixture of broad-spectrum antibiotics (ABX) in the drinking water, a protocol which reduces the amounts of SCFAs in the colon.^3^ This did not alter the number or frequency of total cDCs in the colon (Fig. 1c, d).

Colonic cDC can be divided into four populations based on their CD11b and CD103 expression, with the CD103^+^CD11b^-^ cells belonging to the cDC1 lineage, while the CD103^-^CD11b^+^ and double positive (DP) CD103^+^CD11b^+^ cells are cDC2. The small CD103^-^CD11b^-^ “double negative” (DN) population is a heterogeneous mixture of poorly defined cDC which may contain developing cells cDC precursors.^21^ We observed that while there were few significant changes in the DC subset frequencies (Fig. 1f), a reduction in the number and frequency in the presence of butyrate was seen in the CD11b^+^CD103^-^ cDC2 populations (Fig. 1g). The overall cellularity followed a similar trend, but did not reach statistical significance (Fig. S3a). Note that the data presented here are pooled from 7 individual experiments, but the effects were not consistent across all 7 experiments and in some we observed that butyrate supplementation reduced CD11b^+^CD103^-^ frequency and cell count, while in other experiments this was not the case (Fig. S3b). Together these data indicate that while ABX and butyrate may influence cDC populations in the colon, the effects *in vivo* are variable, perhaps reflecting differences in the microbiota of different mouse batches.

### Butyrate administration *in vivo* alters the ability of cDC to stimulate T cells

**Fig. 2:**
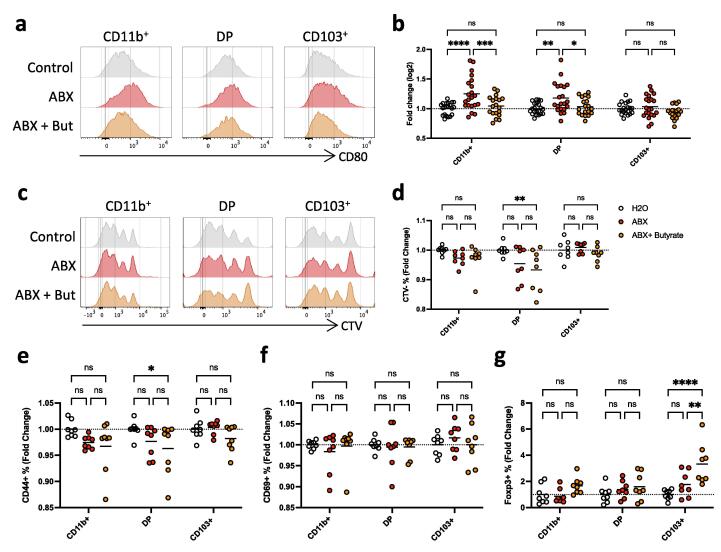
Effects of butyrate on antigen presenting activity of colonic cDC *in vivo.* A) Histograms showing CD80 expression level on cDC susbets in control mice, in mice treated with antibiotic cocktail (ABX) and in mice treated with antibiotics and supplemented with 200 mM butyrate (ABX+Butyrate). B) CD80 expression quantified as fold change of MFI (Mean Fluorescent Intensity) calculated based on control group. cDC were identified as live, single, CD45+, CD3-, B220-, CD64-, MHCII+and CD11c + . Single CD11b^+^ = CD11b^+^CD103^-^, DP=CD11b^+^CD103^+^ double positive and single CD11b^-^CD103^+^ (CD103^+^). C) Histograms showing levels of cell trace violet (CTV) expression by naïve OTII CD4^+^ T cells cultured at a 10:1 ratio for 3 days with OVA-pulsed cDC isolated from colonic LP of control mice, or of mice treated with antibiotic cocktail (ABX) or of mice treated with antibiotics and supplemented with 200 mM butyrate (ABX+Butyrate) D) Fold change of proportions of CTV^-^ of OT-II CD4^+^ T cells after 3 days of co-culture with OVA-pulsed colonic cDC populations calculated base don control group. E-G) Fold change of proportions of naïve OTII CD4^+^ T cells expressing CD44 (E), CD69 (F) and Foxp3 (G) after 3 days of co-culture with OVA-pulsed colonic cDC populations calculated base don control group. Cells pre-gated on live, single, CD45^+^, B220^-^, CD3^-^, CD64^-^, CD11c^+^, MHC^+^ cells(A-B). All T cells pre-gated on live, single, CD3^+^, CD4^+^ cells (C-G). Data shown are pooled from 3 independent experiments with n = 3–5 (A, B) or are pooled from 2 independent experiments with n = 4–5 (C-G). *p < 0.05, **p < 0.01, ***p < 0.001,****p < 0.0001 as assessed by two-way ANOVA with Šídák’s post-test correction for multiple comparisons. ns = not significant.

A hallmark of cDC function is their ability to stimulate the proliferation and differentiation of naïve T cells. To determine if butyrate affected this property of cDC, we first assessed co-stimulatory molecule expression on the colonic cDC subsets in control animals, SCFA-depleted ABX treated animals and butyrate supplemented animals. To overcome the variation between the experiments that we had observed previously, we normalised the data as a fold change relative to the control group. This showed that ABX treatment significantly increased the expression of CD80 by both the CD11b^+^CD103^-^ and CD11b^+^CD103^+^ subsets of cDC2 and this was restored by butyrate supplementation (Fig. 2a,b). CD86 expression was not altered regardless of treatment, (Fig. S3c).

**Fig. 3:**
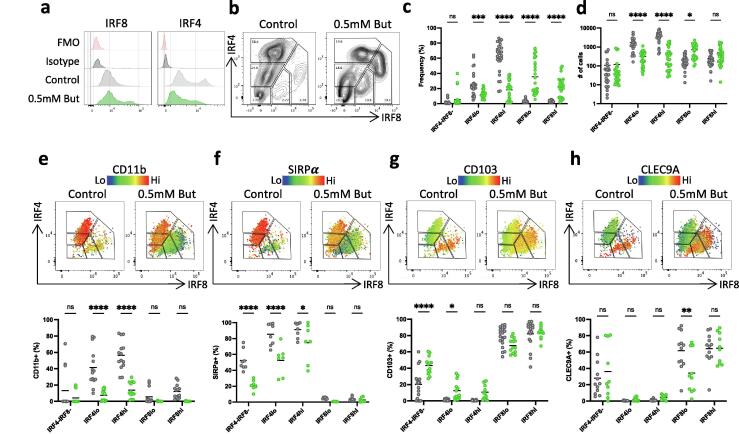
Effects of butyrate on cDC development *in vitro*. A) Histograms and B) contour plots (B) illustrating IRF8 and IRF4 expression by cells generated from BM derived pre-cDC cultured for 4 days in complete medium containing 400 ng/mL Flt3L and 20 ng/mL GM-CSF (control) or with 400 ng/mL Flt3L and 20 ng/mL GM-CSF supplemented with 0.5 mM butyrate, together with isotype and FMO controls. C) Proportions and D) numbers of IRF4 and IRF8 expressing subsets in control and butyrate treated cultures. Heatmap dot plots (upper panels) and proportional expression (lower panels) of E) CD11b, F) SIRPa, G) CD103 and H) CLEC9A within IRF4 and IRF8 defined subsets in control and butyrate treated cultures. All samples pre-gated on live, single, CD45^+^, CD11c^+^, MHC^+^ cells. Data shown are pooled from 5 independent experiments with n = 4–6 each (C, D), or from 3 to 4 independent experiments with n = 3–5 each (E-G). Each data point represents one technical replicate with bars representing the means. *p < 0.05, **p < 0.01, ****p < 0.0001 as assessed by two-way ANOVA with Šídák’s post-test correction for multiple comparisons. ns = not significant.

Next, we assessed the ability of colonic cDC to stimulate T cells *in vitro*, by pulsing FACS-purified colonic cDC populations with ovalbumin and then co-culturing them with purified naive, cell trace violet (CTV) labelled, OVA-specific CD4^+^ T cells. This showed that CD11b^+^CD103^+^ DP cDC2 from butyrate treated mice had a reduced capacity to stimulate T cells, as assessed by proliferation measured by CTV dilution and induction of CD44 expression (Fig. 2c-e). However, butyrate treatment did not alter the ability of these cDC2 to induce CD69 expression on the T cells (Fig. 2f), nor were the stimulatory properties of the other DC populations altered by butyrate. It should also be noted that although CD11b^+^CD103^-^ cDC2 from mice treated with ABX alone showed similar properties to DP cDC2 in co-culture, none of these effects were statistically significant. Finally, we assessed the ability of the various DC populations to induce FoxP3^+^ T regulatory (Treg) cells in the co-culture. Interestingly, this showed that CD11b^-^CD103^+^ cDC1 from mice treated with ABX and supplemented with butyrate had an increased ability to drive Foxp3^+^ Tregs *in vitro*, whereas none of the treatments altered the generation of FoxP3^+^ Tregs by the cDC2 subsets (Fig. 2g).

When examining the effects of butyrate on cDC composition *in vivo*, we found considerable variability in the function of cDC isolated from the colon of mice supplemented with butyrate. To try and overcome this problem, we repeated the study of T cell priming activity using mice treated with butyrate and ABX for 14 instead of 7 days (Figure S4). Under these conditions, addition of butyrate significantly decreased the ability of CD11b^+^CD103^-^ cDC2 to drive the proliferation of CD4^+^ T cells, with a trend towards decreased expression of CD69 and CD44 (Fig. S4a-c). CD11b^+^CD103^+^ cDC2 from butyrate treated mice also showed a decreased ability to induce the expression of CD44 and CD69 by T cells (Fig. S4a-c). In parallel, CD4^+^ T cells cultured with butyrate treated CD11b^+^CD103^-^ cDC2 showed significantly increased expression of Foxp3 expression, with a similar trend seen with CD11b^+^CD103^+^ cDC2 from butyrate treated mice (Fig. S4d). Butyrate treatment for 14 days had no significant effects on the T cell priming effects of cDC1 (Fig. S4a-d).

**Fig. 4:**
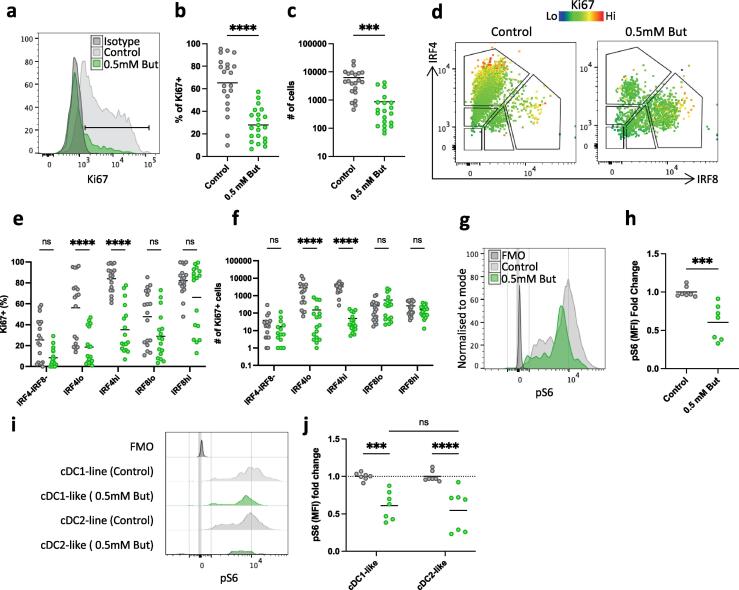
Effects of butyrate on the proliferation of *in vitro* generated cDC. A) Histogram illustrating Ki67 expression by cells generated from BM derived pre-cDC cultured for 4 days in complete medium containing 400 ng/mL Flt3L and 20 ng/mL GM-CSF (control) or with 400 ng/mL Flt3L and 20 ng/mL GM-CSF supplemented with 0.5 mM butyrate, together with isotype control. B) Proportions and C) numbers of Ki67^+^ cells in the presence or absence of butyrate. D) Heatmap dot plot showing Ki67 expression within IRF4 and IRF8 defined subsets. E) Proportions and F) numbers of Ki67^+^ cells amongst IRF4 and IRF8 defined subsets. G) Histogram illustrating phosphorylated ribosomal protein S6 (pS6) staining and H), mean fluorescence intensity (MFI) of pS6 staining in the presence or absence of butyrate. I) Histogram illustrating pS6 staining in cDC1-like (CD103^hi^) and cDC2-like (CD11b^+/h)^ cells and J) mean fluorescence intensity (MFI) of pS6 staining in the presence or absence of butyrate. All samples pre-gated on live, single, CD45^+^, CD11c^+^, MHC^+^ cells. Data shown are pooled from 4 independent experiments with n = 4–5 each (A-F) or from 2 independent experiments with n = 3–4 each (G-J)). Each data point represents one technical replicate with bars representing the means. ***p < 0.001, ****p < 0.0001 as assessed by Student’s *t*-test (B, C, H) or two-way ANOVA with Šídák’s post-test correction for multiple comparisons(E, F,J). ns = not significant.

Together, these data show that as well as altering the relative abundance of colonic cDC subsets, butyrate treatment *in vivo* can modify their phenotype and ability to stimulate CD4^+^ T cells *in vitro*. The *in vivo* effects of ABX and butyrate in our experiments were variable, perhaps reflecting the complexity of the intact intestine and its microbiota. However, combining these results together those seen in GF mice, and in the literature, we reasoned that butyrate may directly influence cDCs. Therefore, to gain a clearer understanding of this complex biology, we aimed to study the effects of butyrate on cDCs under more controlled conditions *in vitro*.

### Butyrate affects pre-cDC development in a lineage-dependent manner

We first examined the direct effects of butyrate on cDC development *in vitro* by establishing a method for generating intestine-like cDC in which highly purified bone marrow (BM)-derived pre-cDCs were cultured with the DC growth factors Flt3L and GM-CSF. Pre-cDCs were identified as CD3^-^ CD19^-^ NK1.1^-^ CD11b^-^ MHCII^-^ CD11c^+^ B220^-^ CCR9^-^ CD135^+^ as described previously^22^ (Figure S5) and after 4 days of culture, cells were assessed for their expression of mature cDC markers (Figure S6). At this time, all viable CD45^+^ cells expressed CD11c and MHCII, but not the macrophage lineage marker CD64; in addition, they lacked expression of the cDC precursor markers Siglec-H and Ly6C, together indicating the generation of *bona fide* differentiated cDC. The majority of the cultured cells expressed some levels of both CD11b and CD103, reminiscent of the phenotypes of the cDC subsets found in the murine intestine.^23^, ^24^, ^25^ Further characterisation of these *in vitro*-generated cells enabled them to be clearly divided into cDC1-like and cDC2-like populations (Figure S7). First, when we identified cells based on differences in their CD11b and CD103 expression (S7a-d), we found that the CD103^hi^ population is also positive for CLEC9A (S7a) and transcription factor IRF8 (S7b), like cDC1 *in vivo*. Conversely, the CD11b^+/hi^ population expressed high levels of SIRPα and IRF4, but not CLEC9A or IRF8, resembling cDC2 *in vivo.* Similar separation could be seen when SIRPα and CLEC9A were used to identify the populations, with CD103 and IRF8 expression being highest in the CLEC9^hi^ population (S7e,f), while CD11b and IRF4 were highest in the SIRPα^+/hi^ cDCs (S7g,h). Interestingly, the clearest separation was obtained when the cells were gated on the basis of IRF4 and IRF8 expression, where the IRF4^+^ cells expressed the highest levels of SIRPα and CD11b (S7e, k), while the IRF8^+^ cells expressed the highest levels of CD103 and CLEC9A (S7j,l). We have been unable to detect expression of the cDC1 marker XCR1 by flow cytometry in these *in vitro* cultures (Supplementary Fig. 7n).

Together these results indicate that the *in vitro* culture system generates populations of cDC1-like and cDC2-like cells similar to those seen *in vivo*, including the subset of CD103^+^ cDC2 that is unique to the intestine. However, one difference was that we could not detect expression of CD101 by any of our cultured DCs (S7m), despite this being highly expressed by CD103^+^ cDC2 and by some CD103^-^ cDC2 *in vivo*.^26^ Thus, while our *in vitro* system generates cells resembling cDC1 (CLEC9A^+^, CD103^+^, IRF8^+^) and cDC2 (SIRPα^+^, CD11b^+^, IRF4^+^), it does not completely replicate all intestinal cDC populations.

We first attempted to establish an optimal concentration of butyrate to use with developing cDC *in vitro* and found that concentrations above 0.5mM caused significant amounts of cell death (Figure S8a-c). cDC2-like cells were more sensitive to this effect than cDC1-like cells (Figure S8d-f). At 0.5mM butyrate effects on viability were not significant (Figure S8f), thus we used this concentration in our further work.

Cells resembling the cDC1 and cDC2 lineages were identified *in vitro* by analysing their characteristic transcription factors IRF8 (cDC1) and IRF4 (cDC2).^27^ Analysis of the cDC generated under control conditions using these markers revealed five distinct populations, the largest of which expressed high levels of IRF4 alone (IRF4^hi^). In addition, there was a somewhat smaller population expressing intermediate levels of IRF4 alone (IRF4^lo^) as identified compared with isotype and fluorescence-minus-one (FMO) controls (Fig. 3a). Similarly, two small populations of IRF8^hi^ and IRF8^lo^ cells were also identified as well as a final population that expressed neither IRF4 nor IRF8 (IRF4^-^IRF8^-^) (Fig. 3b). Addition of butyrate led to large decreases in both the proportions and numbers of IRF4^hi^ cells, together with a significant reduction in the numbers of IRF4^lo^ cells. In contrast, butyrate caused significant increases in the proportions of IRF8^lo^ and IRF8^hi^ cells in the cultures, and a significant increase in the number of IRF8^lo^ cells (Fig. 3b-d). These effects of butyrate on cDC2 and cDC1 were confirmed in a dose–response experiment (Figure S8g-i). Importantly, the cells generated in the presence of butyrate retained their CD64^-^Ly6C^-^MHCII^+^CD11c^+^ phenotype, consistent with *bona fide* differentiated cDC (Figure S9a). Thus, butyrate has distinct effects on cDC1 and cDC2 development *in vitro*, predominantly inhibiting the cDC2 lineage development while having lesser impact on the cDC1 lineage cells.

To confirm these findings, we analysed the effects of butyrate on the expression of the cDC surface markers CD103, CLEC9A, CD11b and SIRPα. Confirming their cDC2 identity, the IRF4^hi^ and IRF4^lo^ populations expressed high levels of CD11b and SIRPα, but lacked expression of CD103 or CLEC9A, which were restricted to the IRF8^hi^ and IRF8^lo^ populations that lacked expression of CD11b and SIRPα (Fig. 3e-h). Together these results validate our use of IRF4 and IRF8 expression for identifying cDC2 and cDC1 respectively. Consistent with its ability to reduce the development of IRF4^+^ cells, butyrate treatment led to a significant decrease in the proportion of CD11b^+^ and SIRPa^+^ cDC2 (Fig. 3e, f). CD103 expression did not change amongst the IRF8^lo^ and IRF8^hi^ populations after treatment with butyrate, but there were significant increases in CD103 expression by the IRF4^-^IRF8^-^ and IRF4^lo^ populations (Fig. 3g, h). Finally, CLEC9A expression was slightly decreased in the IRF8^lo^ population treated with butyrate, but there was no effect on the IRF8^hi^ cells (Fig. 3g, h). Again, none of the cells expressed XCR1 (Figure S9b).

### Butyrate alters pre-cDC proliferation *in vitro* in a lineage-dependent manner

We next explored whether the selective inhibition of cDC2 development by butyrate reflected effects on proliferation, by assessing Ki67 expression by the cells generated from pre-cDC *in vitro*. This showed that the overall frequency and number of Ki67^+^ cells were reduced in cells cultured in the presence of butyrate compared with cells cultured with Flt3L + GM-CSF alone (Fig. 4a-c). Closer analysis showed that this reflected decreased Ki67 expression by the IRF4^lo^ and IRF4^hi^ populations, whereas there was no significant effect on the proliferation of the IRF8^+^ populations (Fig. 4d-f). Together, these data confirm that butyrate modifies pre-cDC development, with a preferential effect on proliferation and differentiation of the CD11b^+^ SIRPα^+^ IRF4^+^ cDC2-lineage compared with the CD103^+^ CLEC9A^+^ IRF8^hi^ cDC1-lineage.

Butyrate has been shown to influence myeloid cell development by modulating cell metabolism and inhibiting mTOR signalling^5^ and as mTOR is a crucial component of proliferation, we analysed mTOR activity in the developing cDC by staining for phosphorylated ribosomal protein S6, a downstream target of mTOR.^28^ This revealed a significant decrease of pS6 staining in the presence of butyrate, suggesting a metabolic shift towards decreased mTOR activity. To examine if the effects on pS6 expression were selective to a specific subset cDC, we had to return separating cDC1 and cDC2 on the basis of CD11b and CD103 expression, as the method used to analyse phosphorylation by phospho-flow is not compatible with intracellular staining of IRF4 and IRF8. This showed that butyrate reduced expression of pS6 by both DC1-like and cDC2-like cells (Fig. 4i,j).

Finally, having identified these changes *in vitro* we returned to the *in vivo* model, assessing Ki67 and pS6 levels after butyrate supplementation of ABX treated mice (Figure S10a,b). Despite variability between animals, while Ki67 expression was not changed in the CD11b^+^ single positive cDC2, DP cDC2 and CD103^+^ cDC1 from ABX treated mice showed increased expression of Ki67 upon ABX treatment and this change was somewhat disrupted by additional supplementation with butyrate (Figure S10a). This is consistent with our *in vitro* observations. However, when we assessed mTOR activity in colonic cDC by staining for pS6, unexpected changes were observed. We identified that ABX treatment alone reduced the overall pS6 staining in both subsets of cDC2, but not in the CD103^+^ cDC1. This could be explained by reduced microbial presence and thus less TLR stimulation and antigen availability leading to subsequent reduced mTOR phosphorylation.^29^ Upon addition of butyrate this effect remained in both cDC2 populations (Figure S10b), indicating that addition of butyrate was unable to significantly strengthen this ABX-induced effect on pS6 levels in cDC2. Therefore, while our *in vitro* observations of pS6 replicate similar observations of the effects of butyrate on macrophages^5^, *in vivo* the ABX treatment alone likely masked the possible butyrate effect. Together these data indicate that butyrate has a clear effect on cDC proliferation and mTOR activity *in vitro* that can also be partly observed in our *in vivo* model.

### The effects of butyrate on cDC are independent of GPR43 or other GPCR

**Fig. 5:**
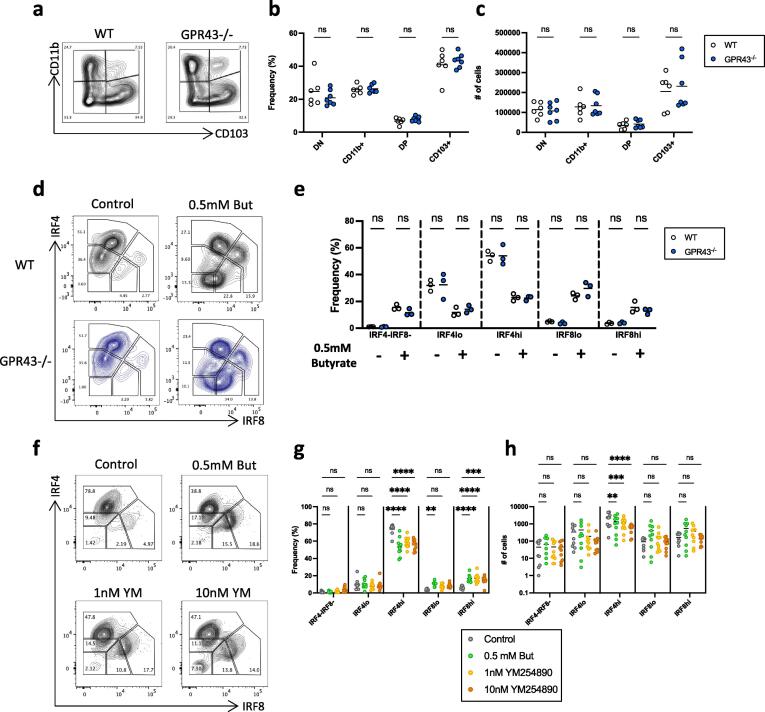
Role of GPCR in development of cDC *in vivo* and *in vitro*. A) Representative FACS plots of colonic LP cDC subsets based on CD11b and CD103 expression in GPR43^-/-^ mice and WT littermates. B) Frequency amongst total cDC and C) numbers of colonic LP cDC subsets. DN=CD11b^-^CD103^-^ double negative, single CD11b^+^ = CD11b^+^CD103^-^, DP=CD11b^+^CD103^+^ double positive and single CD11b^-^CD103^+^ (CD103^+^). D) Contour plots and E), proportions of IRF4 and IRF8 expressing subsets of *in vitro* generated cDC from GPR43^-/-^ or WT littermates in control and butyrate treated cultures. F) Contour plots, G) proportions and H) absolute numbers of IRF4 and IRF8 expressing subsets of cDC generated in Flt3L and GM-CSF alone (control) or with the addition of 0.5 mM butyrate in the presence or absence of the pan-GPCR inhibitor YM254890 (1 nM,10 nM). Ex-vivo cells pre-gated on live, single, CD45^+^, B220^-^, CD3^-^, CD64^-^, CD11c^+^, MHC^+^ cells.(A-C). All *in vitro* samples pre-gated on live, single, CD45^+^, CD11c^+^, MHC^+^ cells (D-H). Data shown are pooled from 2 independent experiments with n = 2–4 each (A-C), from one experiment with n = 3 (D) or are pooled from 2 independent experiments with n = 4–6 (E-H). Each data point represents one mouse (A-C) or an individual technical replicate (D-H) with bars representing the means. *p < 0.05, **p < 0.01, ***p < 0.001, ****p < 0.0001 as assessed by two-way ANOVA with Šídák’s post-test correction for multiple comparisons. ns = not significant.

An important way by which butyrate can exert its cellular effects is via the G protein coupled receptor GPR43.^30^ To examine if this was involved in the changes in cDC populations caused by butyrate, we first quantified colonic cDCs in steady state mice lacking GPR43. No differences were observed in the frequencies or absolute numbers of colonic cDC populations in these mice compared with wild-type littermate controls (Fig. 5a-c). We then cultured pre-cDCs from GPR43^-/-^ mice and assessed their IRF4 and IRF8 expression in the presence or absence of butyrate (Fig. 5d). As before, culture with butyrate caused significant decreases in the populations of wild-type cDC expressing IRF4 but had no significant effect on the IRF8-expressing cells. Identical effects of butyrate were seen with GPR43^-/-^ cDC (Fig. 5e). Finally, to understand whether butyrate may be signalling though other GPCRs, we used the pan-GPCR inhibitor YM254890 in the pre-cDC cultures (Fig. 5f).^31^, ^32^ 1 or 10nM concentration of the GPCR inhibitor had no effect on the ability of butyrate to alter the frequencies of the IRF4 and IRF8-expressing populations (Fig. 5g), nor on the total cell count (Fig. 5h). Together these data indicate that the effects of butyrate on the cDC2-lineage are not GPCR-dependent.

### The effects of butyrate on cDC development can be replicated by inhibition of HDAC3

As the effect of butyrate on developing cDC do not depend on GPCR signalling, we tested whether this effect might result from HDAC inhibition. Normally, HDACs remove acetyl groups from chromatin tails and thus facilitate methylation of these residues. Acetylation of histone residues is often associated with higher gene expression, while methylation can be a sign of gene silencing. As butyrate increases acetylation levels in macrophages ^5^ other non-lymphoid cells,^15^ we hypothesised that butyrate may affect developing cDCs through HDAC inhibition. To assess the possible role of butyrate as an HDAC inhibitor in cDCs, acetylation of histone 3 (Lys27) (H3K27ac) and triple methylation of histone 3 (Lys27) (H3K27me3) were measured using antibodies and flow cytometry as previously described by Schulthess et al., 2019^5^.

**Fig. 6:**
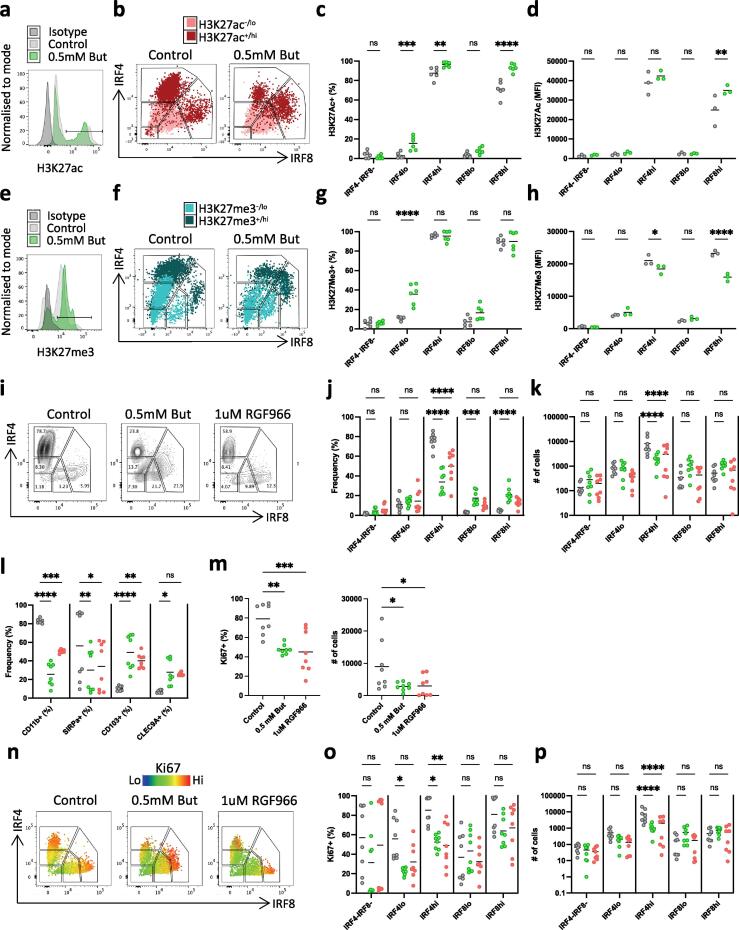
Role of HDAC inhibition in effects of butyrate on cDC development *in vitro*. A) Histogram illustrating expression of H3K27ac by cDC generated in Flt3L and GM-CSF alone (control) or with the addition of 0.5 mM butyrate, together with isotype control. B) Dot plot overlay of H3K27ac^-/lo^ and H3K27ac^+/hi^ cells within subsets defined by IRF4 and IRF8 C) Proportions and D) mean fluorescence intensity (MFI) of H3K27ac^+^ cells within subsets defined by IRF4 and IRF8. E) Histogram illustrating expression of H3K27me3 by cDC generated in Flt3L and GM-CSF alone (control) or with the addition of 0.5 mM butyrate, together with isotype control. F) Dot plot overlay of H3K27me3-/lo and H3K27me3+/hi cells within subsets defined by IRF4 and IRF8 G) Proportions and H) mean fluorescence intensity (MFI) of H3K27me3^+^ cells within subsets defined by IRF4 and IRF8. I) Contour plots, J) proportions and K) absolute numbers of IRF4 and IRF8 expressing subsets of cDC generated in Flt3L and GM-CSF alone (control) or with the addition of 0.5 mM butyrate, or with 1 µM of the HDAC3 agonist RGF966. L) Proportions of *in vitro* generated cDC expressing CD11b, SIRPa, CD103 or CLEC9A in control cultures or after addition of butyrate or RGF966. M) Proportions of *in vitro* generated cDC expressing Ki67 in control cultures or after addition of butyrate or RGF966. N) Overlay dot plots showing Ki67 expression by IRF4 and IRF8 defined subsets of cDC generated in control cultures or after addition of butyrate or RGF966. O) Proportions and P) numbers of Ki67^+^ cells in IRF4 and IRF8 defined subsets. All *in vitro* samples pre-gated on live, single, CD45^+^, CD11c^+^, MHC^+^ cells. Data shown are pooled from 2 independent experiments with n = 3–4 each. Each data point represents one technical replicate with bars representing the means. *p < 0.05, **p < 0.01, ***p < 0.001, ****p < 0.0001 as assessed by Student’s *t*-test (M) or a two-way ANOVA with Šídák’s post-test correction for multiple comparisons (C, D, G, H, J, K L, O, P). ns = not significant.

When cells were analysed for H3K27Ac (Fig. 6a), based on their IRF4 and IRF8 expression, the most highly acetylated cells co-localised with the IRF4^hi^ and IRF8^hi^ populations (Fig. 6b). Increase of H3K27Ac correlates with increased maturation of human myeloid progenitors (Grassi et al., 2018) and with stem cell differentiation (Zhang et al., 2020). This is consistent with the higher expressing IRF4 and IRF8 cells representing more highly differentiated cDCs. When butyrate was added *in vitro,* the proportions of acetylated IRF4^lo^, IRF4^hi^ and IRF8^hi^ cells increased (Fig. 6c). A small increase in the mean acetylation level was also observed in the IRF8^hi^ population (Fig. 6d). Thus, in the presence of butyrate, while the number of mature IRF4^hi^ cells is decreased (Fig. 4), the proportion of acetylated cells increases, and the level of acetylation increases in the IRF8^hi^ population. These data are consistent with the hypothesis that butyrate increases histone acetylation levels by inhibiting HDACs.

**Fig. 7:**
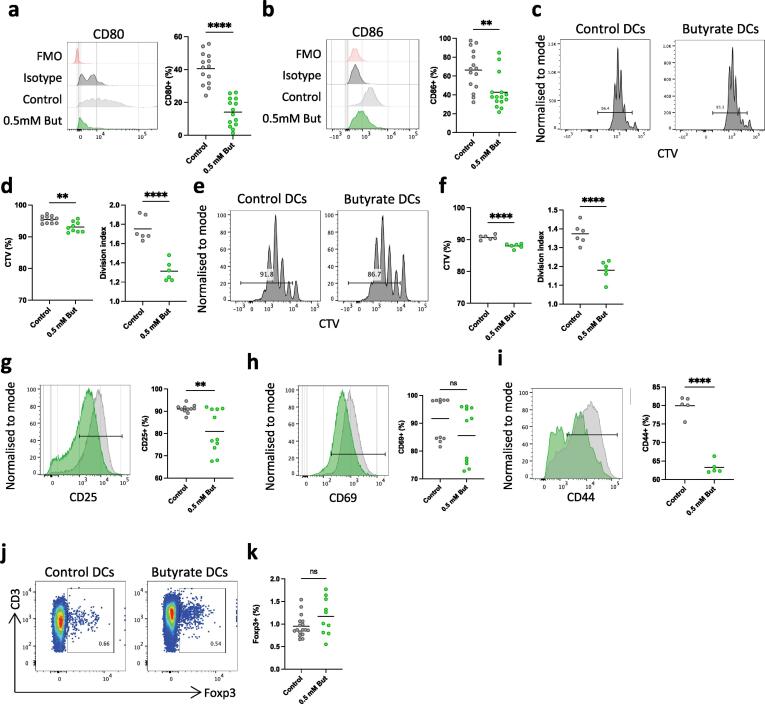
Effects of butyrate on antigen presenting activity of *in vitro* generated cDCs. A) Histogram showing CD80 expression by cells generated from BM derived pre-cDC cultured for 4 days in complete medium containing 400 ng/mL Flt3L and 20 ng/mL GM-CSF (control) or with 400 ng/mL Flt3L and 20 ng/mL GM-CSF supplemented with 0.5 mM butyrate, together with isotype and FMO controls (left panel) and proportions of cells expressing CD80 (right panel). A) Histogram showing CD86 expression by cells generated from BM derived pre-cDC cultured for 4 days in complete medium containing 400 ng/mL Flt3L and 20 ng/mL GM-CSF (control) or with 400 ng/mL Flt3L and 20 ng/mL GM-CSF supplemented with 0.5 mM butyrate, together with isotype and FMO controls (left panel) and proportions of cells expressing CD80 (right panel). C) Histograms showing levels of cell trace violet (CTV) expression by naïve OTI CD8^+^ T cells cultured at a 10:1 ratio for 3 days with OVA-pulsed cDC generated in control cultures *in vitro* or in the presence of butyrate. D) Proportions of CTV^-^ (left panel) and division index (right panel) of OT-I CD8^+^ T cells after 3 days of co-culture with OVA-pulsed control or butyrate treated cDC. E) Histograms showing levels of cell trace violet (CTV) expression by naïve OTII CD4^+^ T cells cultured at a 10:1 ratio for 3 days with OVA-pulsed cDC generated in control cultures *in vitro* or in the presence of butyrate. F) Proportions of CTV^-^ (left panel) and division index (right panel) of OT-II CD4^+^ T cells after 3 days of co-culture with OVA-pulsed control or butyrate treated cDC. G-I) Representative histograms (left panels) and proportions (right panels) naïve OTII CD4^+^ T cells expressing CD25 (E), CD69 (F) and CD44 (G) after 3 days of co-culture with OVA-pulsed control or butyrate treated cDC. J) Representative dot plots showing Foxp3 expression and K), proportions of OTII CD4^+^ T cells expressing FoxP3. All *in vitro* samples pre-gated on live, single, CD45^+^, CD11c^+^, MHC^+^ cells (A,B). All T cells pre-gated on All *in vitro* samples pre-gated on live, single, CD3^+^, CD4^+^ cells. Data shown are pooled from 4 independent experiments with n = 3–5 (A, B), or are representative of 2 independent experiment with n = 6 (C, D), or are pooled from 2 independent experiments with n = 5–12 (E-I). Each data point represents one technical replicate with bars representing the means. Data *p < 0.05, **p < 0.01, ****p < 0.0001 as assessed by Student’s *t*-test (A,B D, I, J) or two-way ANOVA with Šídák’s post-test correction for multiple comparisons (F,G). ns = not significant.

H3K27me3 is a histone modification associated with overall deacetylation,^33^ which decreases in response to butyrate ^5^. Therefore, this methylation pattern should be opposite to the acetylation pattern. When antibody staining was used to measure H3K27me3 (Fig. 6e) we observed, similar to acetylation, that there were no significant differences between the control and butyrate treated cells apart from an increase in staining of the IRF4^lo^ cells, consistent with these being more immature (Fig. 6f, g). Taken together with the observation that IRF4^lo^ cells also increased the proportion of acetylated cells, this could possibly indicate a population of cells that partly resembles IRF4^hi^ cells, but their development from IRF4^lo^ into IRF4^hi^ cells has been inhibited, as illustrated by the decrease on Ki-67 staining in them (Fig. 4). When mean methylation levels were quantified, this revealed a significant decrease in H3K27me3 staining in both IRF4^hi^ and IRF8^hi^ cells (Fig. 6h). This loss of methylation is again consistent with HDAC inhibition by butyrate in these populations. These changes are not subset specific and thus do not explain how butyrate can have a selective effect on cDC2s over cDC1s. However, they do indicate epigenetic modulation after butyrate treatment, showing that butyrate can affect HDAC inhibition in cDCs.

We also assessed histone modifications in the *in vivo* model. We observed a significant increase of H3K27ac in colonic cDC from ABX treated animals compared with controls, and butyrate supplementation did not alter this (Figure S10c). In turn, no changes in H3K27me3 were observed *in vivo* colonic cDC populations (Figure S10d) This increase in acetylation might reflect altered gene expression patterns in response to ABX rather than overall HDAC activity. We conclude that assessment of total H3K27ac or H3K27me3 is not precise enough to assess cell and/or gene specific HDAC inhibition.

As butyrate has been previously described to be a specific HDAC3 inhibitor,^5^, ^15^ we next compared the effects of butyrate with those of a known HDAC3 inhibitor, RGF966.^34^ We used RGF966 at a previously described bioactive concentration of 1mM.^35^ These experiments showed that RGF966 and butyrate had an identical ability to reduce the proportion and number of IRF4^hi^ cells, while the numbers of IRF8^lo^ and IRF8^hi^ cells were unchanged in both conditions (Fig. 6i-k). Furthermore, both RGF966 and butyrate led to comparable reductions in the proportions of CD11b^+^ and SIRPα^+^ cells, with corresponding increases in the proportion of CD103^+^ and CLEC9A^+^ cells (Fig. 6l). Finally, both butyrate and RGF966 also reduced both the proportion and the number of total Ki-67^+^ cells (Fig. 6m), an effect which was restricted to the IRF4^lo^ and IRF4^hi^ populations, with no significant changes in the proliferation of the IRF8^lo^ and IRF8^hi^ populations (Fig. 6n-p). Together, these findings indicate that the effects of butyrate on cDC development are independent of GPCRs but may reflect its ability to act as an HDAC3 inhibitor.

### Effects of butyrate on the ability of cDC to activate CD4^+^ and CD8^+^ T cells

As butyrate altered the phenotype and potential maturation of the *in vitro* generated cDCs, we examined how this might affect their ability to activate T cells *in vitro*. To do this, we first assessed the expression of co-stimulatory molecules and found that most cells cultured in Flt3L + GM-CSF alone expressed CD80 and CD86 to some extent, with both markers being reduced almost to background in the presence of butyrate (Fig. 7a, b). To test their antigen presenting cell activity, the *in vitro* generated cells were pulsed with ovalbumin protein and then cultured with purified naive, cell trace violet (CTV) labelled, OVA-specific CD8^+^ or CD4^+^ T cells and T cell proliferation and phenotype were assessed using flow cytometry. After 3 days of culture, both CD8^+^ and CD4^+^ T cells cultured with control cDCs showed considerable dilution of CTV indicating antigen-specific proliferation.

cDC2 are often described to drive CD4^+^ T cell responses *in vivo*, while cDC1 can drive both CD4^+^ and CD8^+^ T cell responses. Thus, we hypothesised that as the DC1-lineage cells are not altered as much as DC2-like cells *in vitro* during butyrate treatment, we would only see effects on priming of CD4^+^ T cells and of CD8^+^ T cells. Contrary to this hypothesis, both CD4^+^ and CD8^+^ T cell responses were significantly reduced when butyrate treated cDC were used (Fig. 7c-f) perhaps reflecting the fact that cDC2 have been shown to induce some CD8^+^ T cell proliferation *in vitro*.^20^ Alternatively, while butyrate supplementation might not alter the DC1-like cell abundance to the same level as DC2-like cells, it could affect their CD8^+^ T cell cross-priming abilities. This would be in line with the overall decreased co-stimulatory molecules. CD4^+^ T cells that were co-cultured with butyrate-treated cDC also had reduced expression of the activation markers CD25, CD69 and CD44 (Fig. 7g-i), although these effects on CD4^+^ T cells were not accompanied by increased induction of Foxp3^+^ Treg (Fig. 7j, k).

Together these data show that butyrate reduces the expression of costimulatory molecules by cDCs *in vitro* and *in vivo*, leading to a reduced ability to drive activation and proliferation of naïve T cells *in vitro* and to some extent also *in vivo.*

## Discussion

We first observed that *in vivo* manipulation of microbiota derived SCFA in the intestine may alter intestinal cDC population abundance, phenotype and function. As it was unclear if butyrate has a direct effect on cDC and what molecular mechanisms may be behind this observation, we then devised an *in vitro* system to study this in more detail. By purifying pre-cDC cells instead of culturing whole bone marrow, we avoided the previously described caveat to the *in vitro* generation of BMDC, where large proportion of the cells generated are of monocyte and macrophage lineage.^36^, ^37^, ^38^ Our method allowed for generation of true cDC-lineage cells that also develop features of intestinal cDC as was evident by the presence of such surface markers as CD11b, CD103, SIRPα and CLEC9A. Of note, these cells lacked expression of XCR1, consistent with the recent findings that XCR1 is not expressed on *in vitro* generated cDC until later time points such as day 7 or 10.^39^, ^40^ This suggests that the cDC generated by our method may be relatively early in their developmental trajectory and later effects remain to be explored. Nevertheless, this novel system allowed us to demonstrate that butyrate directly inhibited the development and proliferation of cDC2-like cells, with fewer clear effects on cells of the cDC1-lineage.

Having identified possible lineage dependent effects of butyrate, we investigated the molecular mechanisms underlying these. Butyrate can signal both through GPCR and directly via HDAC inhibition.^15^, ^41^, ^42^ Neither the absence *in vivo* or inhibition of GPCRs *in vitro* altered cDC populations *in vivo* or affected the ability of butyrate to modulate cDC development *in vitro*. On the other hand, we observed butyrate-mediated changes in histone modifications that are associated with HDAC activity. Furthermore the effects of butyrate on cDC development were replicated by the HDAC3 inhibitor RGF966 and we conclude that HDAC inhibition is likely to be the mechanism by which butyrate alters cDC development. Similar findings have been made for the modulation of macrophage differentiation and function by butyrate.^5^

It remains unclear why butyrate affects the cDC1 and cDC2 lineage cells differentially. A possible explanation may be that cDC1 and cDC2 cells have different metabolic requirements. The ability of butyrate to inhibit HDAC activity in macrophages is associated with mTOR modulation via HDAC inhibition ^5^. As mTOR activity in cDC is subset dependent, this could explain our observations. In particular, mTOR is a positive regulator of HIF1a^43^ which is an upstream regulator of the expression of the cDC2 marker CD11b.^44^ Indeed, it has been shown *in vivo* that deletion of mTOR results in decrease of other CD11b^+^ cells, such as macrophages.^45^ In our hands, butyrate reduced mTOR activity in all subsets of *in vitro* generated cDCs and we would propose that the selective effect of butyrate on cDC2 development may indicate that mTOR signalling may be more important for the development of cDC2 than cDC1. Alternatively, it has been reported that HDAC activity, especially that of HDAC3, contributes to the STAT3 signalling pathway.^46^ As STAT3 signalling is crucial for cDC2 development^44^, ^47^ and is inhibitory to cDC1 development,^48^, ^49^ this could be another way in which butyrate could have a lineage-specific effect.

Colonic cDC isolated from butyrate supplemented animals treated with antibiotics had lower levels of co-stimulatory molecule expression compared with those found after antibiotic treatment alone. However no consistent effects on T cell stimulatory activity could be seen using cDC from mice treated with butyrate *in vivo*, although cDC1 isolated from butyrate supplemented animals increased their capacity to drive Treg development. As it seemed possible that these conflicting results might reflect confounding effects of the other cell types that could be targets of orally administered SCFA, we examined the direct effects of butyrate on the antigen presenting capacity of the cDC generated in our *in vitro* culture model. This showed that butyrate treated cDC had a reduced expression of co-stimulatory molecules and a reduced ability to induce T cell proliferation and activation. Yet, their ability to induce Foxp3 remained unchanged. Together, these data indicate that butyrate may alter cDC development in such a way that their ability to prime T cells is reduced. This could be consistent with the other indications we found that butyrate reduced the maturation of cDC, leading to a relatively immature state. In support of this idea, the abundance of SCFAs *In vivo* correlates with reduced development of effector T cells^11^, ^12^ and immature cDC have been implicated to play an important role in maintaining tolerance to the commensal microbiota.^50^

Altogether, our *in vitro* work offers mechanistic insights into the known association of SCFA with anti-inflammatory immune responses.^9^, ^15^, ^51^ We reveal a novel mechanism by which microbiota-derived SCFA butyrate can shape the composition of cDCs, modulating their development and functions in a lineage-specific manner. This observation not only furthers our understanding of how microbiota-host interactions in the steady state may maintain homeostasis, but also opens possibilities for targeted manipulation of immune cells by altering SCFA abundance in the intestine. Importantly, butyrate has also been shown to have effects on human cDC, thus giving our findings translational potential.^52^, ^53^ Finally, this knowledge may also be beneficial in an age where tailored dietary interventions are beginning to be used to treat immune disorders, such as inflammatory bowel disease.^54^

Methods

### Animals

C57BL/6 mice were purchased from Envigo at 5–6 weeks of age and used for procedures at 7–8 weeks of age. Mice were housed under specific pathogen free conditions at the Common Research Facility, University of Glasgow. OT1.Rag^-/-^ mice were a kind gift from Professor Rose Zamoyska, University of Edinburgh, while OTII CD45.1 mice were a gift from Dr Ed Roberts (Beatson Cancer Research Institute, Glasgow). GPR43^-/-^ KO mice and WT littermates were a gift from Professor Graeme Milligan (University of Glasgow). Age and sex matched animals were used for all experiments and all procedures were carried out under personal and project licences issued by the UK Home Office.

### Manipulation of SCFA abundance *in vivo*

Mice were administered 200mM sodium butyrate (Sigma) for 7 or 14 days in their drinking water. Control animals received normal water. For antibiotic treatment, mice were administered a cocktail of broad-spectrum antibiotics in their drinking water for 7 days. The cocktail consisted of ampicillin (1g/litre) (Sigma), metronidazole (1g/litre) (Sigma), neomycin (1g/litre) (Sigma), gentamicin (1g/litre)(Sigma), and vancomycin (0.5g/litre)(Wockhardt) and was replaced once, after 3 to 4 days.

### Isolation of lamina propria cells

Small intestine (SI) and colon were removed, cut open longitudinally and rinsed in cold PBS, with Peyer’s patches being removed from the small intestines. Intestinal tissue was enzymatically digested as described previously (Webster and Andrusaite, 2019). In brief, the epithelium and mucus were first removed by washing tissues 3 times in warm calcium/magnesium free HBSS containing 2mM EDTA (Gibco) for 15 min at 37°C with shaking at 250rpm. Small intestines were then digested by incubation with 0.5mg/mL collagenase VIII (Sigma), while colons were digested with 0.5mg/mL collagenase V (Sigma), 0.65mg/mL collagenase D (Roche), 1mg/mL Dispase (Gibco) and 30ug/mL DNase (Roche) each for 15–20 min at 37°C with shaking. Digestion was stopped by adding cold complete RPMI medium (RPMI 1640 contained 10% heat inactivated foetal calf serum (FCS), 2mM L-glutamine, 100μm/mL penicillin/streptomycin and 50μm b-mercaptoethanol (all Gibco). Cells were then passed through a 100μm and then a 40μm mesh EASYstrainer (Greiner), before being washed twice with cold complete RPMI medium for 10 min in 4°C at 400g at stored at 4°C before use.

### Generation of cDC from bone marrow precursors *in vitro*

Bone marrow cells were isolated from femurs and tibiae of 3–4 adult mice per experiment. Red blood cells were lysed using ACK lysing buffer (Thermo Fisher) for 1 min and cells were washed with FACS buffer for 10 min at 4°C at 400g. Pre-cDC were then purified by first enriching using a MagniSort^TM^ Magnet (eBioscience) and MagniSort^TM^ SAV Negative Selection Beads (eBioscience) with biotinylated anti-CD3, anti-CD19, anti-Ly6G and anti-B220 at a 1:300 dilution. The resulting cells were then FACS sorted on an BD FACSAria^TM^ III using anti-CD3-BV605(1:100), anti-CD19-BV605 (1:100), anti-NK1.1-PercP-Cy5.5 (1:100) and anti-CD135-biotin in combination with PE-streptavadin (1:200) (all from eBioscience) and anti-CCR9-PE-Cy7 (1:100), anti-B220 (1:200), anti-CD11c-FITC(1:100), anti-CD11b-APC-Cy7 (1:200) and anti-MHCII-BV421 (1:200) (all BioLegend).

Aliquots of approximately 15,000 pre-cDCs were then cultured for 4 days in a total volume of 150μl in flat-bottomed 96-well plates (Costar, #3596) containing complete RPMI medium supplemented with 20ng/mL GM-CSF (Peprotech) and 400ng/mL Flt3L (Peprotech), together with different concentrations of butyrate (Sigma), YM254890 (Tocris) or 1µM RGF966 (Tocris). Cells were cultured at 37°C and 5% CO2.

### Flow cytometry

Aliquots of 5–10 x 10^6^ cells were first assessed for viability staining using eFlour780 (eBioscience) fixable viability dye (1:1000) or 7AAD (Biolegend) viability dye (1:100). Non-specific binding to FC receptors was prevented by staining with anti-mouse CD16/32 (eBioscience) (1:100) for 20 mins at 4°C. Cells were then stained using the FACS antibodies at a 1:200 dilution in FACS buffer (DPBS supplemented with 2mM EDTA (Gibco) and 2% FBS(Sigma)) unless specified otherwise (Table 1) for 30 mins at 4°C. For intracellular staining of nuclear transcription factors and histone modifications, the eBioscience Foxp3 Transcription Factor Staining Buffer Set was used according to the manufacturer’s instructions. In brief, cells were fixed using 200μl fixation solution for 1 h at room temperature before being washed with permeabilization buffer for 5 min in 20°C at 400g. Intracellular staining was performed for 1 hr at room temperature. For intracellular staining of phosphorylated S6 the BioLegend True-Phos Perm Buffer was used according to manufacturer’s instructions. Cells were analysed on BD Fortessa and FlowJo software (BD). Expression of proteins was assessed either as a % positive cells of the parent population or as median fluorescence intensity (MFI).

**Table 1:** Antibodies used in flow cytometric analysis.

**Target**	**Manufacturer**	**Clone/CAT**	**Dilution**
Anti-rabbit IgG (H+L), T(ab')2 Fragment (AF488)	Cell Signalling	#4412	1/200
B220	BioLegend	RA3-6B2	1/200
CCR9	Biolegend	CW-1.2	1/200
CD103	BioLegend	2E7	1/200
CD101	eBioscience	Moushi101	1/200
CD11b	BioLegend	M1/70	1/200
CD11c	BioLegend	N418	1/200
CD135	Biolegend	A2F10	1/200
CD19	BioLegend	6D5	1/200
CD25	BioLegend	PC-61	1/200
CD3	BioLegend	172A	1/200
CD45	BioLegend	30-F11	1/200
CD64	BioLegend	X54-5/7.1	1/200
CD80	BioLegend	16-10A1	1/200
CD86	BioLegend/eBioscience	GL1	1/200
CLEC9A	BioLegend	7H11	1/200
eFlour 780	eBioscience	#65–0865-14	1/1000
H3K27ac	Cell Signalling	D5E4	1/200
H3K27me3	Cell Signalling	C36B11	1/100
IRF4	Biolegend	IRF4.3E4	1/200
IRF8	eBioscience	V3GYWCH	1/200
Ki-67	Biolegend	16A8	1/200
Ly6C	BioLegend	HK1.4	1/200
Ly6G	BioLegend	1A8	1/200
MHCII	BioLegend	M5/114.15.2	1/200
NK1.1	BioLegend	PK136	1/200
pS6	BioLegend	A17020B	1/100
Siglec-F	BioLegend	E50-2440	1/200
Siglec-H	BioLegend	551	1/200
SIRPα	BioLegend	P84	1/200
Streptavidin-PE	BD Pharmingen	#554061	1/200
XCR1	BioLegend	ZET	1/200

### Co-culture of DC and T cells

Naïve OVA-specific CD8^+^ T cells were obtained from the spleen and lymph nodes of Rag^-/-^ OTI TCR transgenic mice. Naïve OVA-specific CD4^+^ T cells were purified from the spleen and lymph nodes of OTII TCR transgenic mice using the MojoSort^TM^ Mouse CD4 Naïve T cell isolation Kit according to the manufacturer’s instructions (Biolegend). Cells were then stained with the CellTrace^TM^ Violet Cell proliferation kit (ThermoFisher) according to the manufacturer’s instructions. FACS-isolated colonic cDC populations or *in vitro* generated DC were pulsed with OVA protein (#SLBS4311, Sigma) at 2mg/mL for 2 h at 37°C and co-cultured with T cells at 1:10 ratio for 3 days in complete RPMI in a total volume of 300μl in round-bottomed 96-well plates (Costar, #3799).

### Statistical analysis

Prism (GraphPad) software was used for all statistical analysis. Data were tested for normal distribution using the Shapiro-Wilk test for normality. Statistical differences were calculated using either one-way ANOVA (with Dunnett’s or Tukey’s post-test for multiple comparisons), two-way ANOVA (with Šídák's post-test for multiple comparison) or Student’s *t* test, with the data being plotted as means and SD. Results were considered significant based on their *p*-values (*p<0.05, **p<0.01, ***p<0.001, ****p<0.0001).

### ScRNAseq dataset analysis

Previously published^16^ data set was accessed through GEO Series accession number GSE137927. Data was clustered and visualised using Seurat as previously described^16^.

### Illustrations

Summary illustration was created using BioRender.com with an academic subscription. Publication license number PO25GQABJP.

### CRediT authorship contribution statement

**Anna Andrusaite:** Writing – review & editing, Writing – original draft, Project administration, Methodology, Investigation, Formal analysis, Data curation, Conceptualization. **Jennifer Lewis:** Methodology, Formal analysis. **Annika Frede:** Formal analysis. **Andrew Farthing:** Data curation, Formal analysis. **Verena Kästele:** Methodology. **Jennifer Montgomery:** Methodology, Formal analysis. **Allan Mowat:** Writing – review & editing, Writing – original draft, Methodology, Conceptualization. **Elizabeth Mann:** Methodology, Conceptualization. **Simon Milling:** Writing – review & editing, Writing – original draft, Supervision, Project administration, Methodology, Investigation, Funding acquisition, Formal analysis, Data curation, Conceptualization.

## Declaration of competing interest

The authors declare that they have no known competing financial interests or personal relationships that could have appeared to influence the work reported in this paper.

## References

[1] Ginhoux F. (2009). The origin and development of nonlymphoid tissue CD103+ DCs. J Exp Med.

[2] Kamath A.T., Henri S., Battye F., Tough D.F., Shortman K. (2002). Developmental kinetics and lifespan of dendritic cells in mouse lymphoid organs. Blood.

[3] Scott N.A. (2018). Antibiotics induce sustained dysregulation of intestinal T cell immunity by perturbing macrophage homeostasis. Sci. Transl. Med..

[4] Ji J. (2016). Microbial metabolite butyrate facilitates M2 macrophage polarization and function. Sci Rep.

[5] Schulthess J. (2019). The Short Chain Fatty Acid Butyrate Imprints an Antimicrobial Program in Macrophages. Immunity.

[6] Trompette A. (2018). Dietary Fiber Confers Protection against Flu by Shaping Ly6c− Patrolling Monocyte Hematopoiesis and CD8+ T Cell Metabolism. Immunity.

[7] Esterházy D. (2016). Classical dendritic cells are required for dietary antigen–mediated induction of peripheral Treg cells and tolerance. Nat Immunol.

[8] Fukaya T. (2023). Gut dysbiosis promotes the breakdown of oral tolerance mediated through dysfunction of mucosal dendritic cells. Cell Rep.

[9] Fachi J.L. (2019). Butyrate Protects Mice from Clostridium difficile-Induced Colitis through an HIF-1-Dependent Mechanism. Cell Rep.

[10] Tan J. (2016). Dietary Fiber and Bacterial SCFA Enhance Oral Tolerance and Protect against Food Allergy through Diverse Cellular Pathways. Cell Rep.

[11] Park J. (2015). Short-chain fatty acids induce both effector and regulatory T cells by suppression of histone deacetylases and regulation of the mTOR–S6K pathway. Mucosal Immunol.

[12] Furusawa Y. (2013). Commensal microbe-derived butyrate induces the differentiation of colonic regulatory T cells. Nature.

[13] Smith P.M. (2013). The Microbial Metabolites, Short-Chain Fatty Acids. Regulate Colonic Treg Cell Homeostasis. *Science*.

[14] Liu H. (2018). Butyrate: A Double-Edged Sword for Health?. Adv Nutr.

[15] Silva L.G., Ferguson B.S., Avila A.S., Faciola A.P. (2018). Sodium propionate and sodium butyrate effects on histone deacetylase (HDAC) activity, histone acetylation, and inflammatory gene expression in bovine mammary epithelial cells1. J Anim Sci.

[16] Kang B. (2020). Commensal microbiota drive the functional diversification of colon macrophages. Mucosal Immunol.

[17] Ando K., Ajchenbaum-Cymbalista F., Griffin J.D. (1993). Regulation of G1/S transition by cyclins D2 and D3 in hematopoietic cells. Proc. Natl. Acad. Sci. U.S.A..

[18] Bagadia P. (2019). An Nfil3–Zeb2–Id2 pathway imposes Irf8 enhancer switching during cDC1 development. Nat Immunol.

[19] Schenk R.L. (2017). Characterisation of mice lacking all functional isoforms of the pro-survival BCL-2 family member A1 reveals minor defects in the haematopoietic compartment. Cell Death Differ.

[20] Cerovic V. (2013). Intestinal CD103− dendritic cells migrate in lymph and prime effector T cells. Mucosal Immunol.

[21] Scott C.L. (2015). CCR2+CD103− intestinal dendritic cells develop from DC-committed precursors and induce interleukin-17 production by T cells. Mucosal Immunol.

[22] Schlitzer A. (2015). Identification of cDC1- and cDC2-committed DC progenitors reveals early lineage priming at the common DC progenitor stage in the bone marrow. Nat Immunol.

[23] Cabeza-Cabrerizo M., Cardoso A., Minutti C.M., Pereira da Costa M., Reis e Sousa C. (2021). Dendritic Cells Revisited. Annu. Rev. Immunol..

[24] Guilliams M. (2016). Unsupervised High-Dimensional Analysis Aligns Dendritic Cells across Tissues and Species. Immunity.

[25] Merad M., Sathe P., Helft J., Miller J., Mortha A. (2013). The Dendritic Cell Lineage: Ontogeny and Function of Dendritic Cells and Their Subsets in the Steady State and the Inflamed Setting. Annu. Rev. Immunol..

[26] Bain C.C. (2017). TGFβR signalling controls CD103+CD11b+ dendritic cell development in the intestine. Nat Commun.

[27] Kim S. (2020). High Amount of Transcription Factor IRF8 Engages AP1-IRF Composite Elements in Enhancers to Direct Type 1 Conventional Dendritic Cell Identity. Immunity.

[28] Yang L. (2014). The mTORC1 effectors S6K1 and 4E-BP play different roles in CNS axon regeneration. Nat Commun.

[29] Snyder J.P., Amiel E. (2019). Regulation of Dendritic Cell Immune Function and Metabolism by Cellular Nutrient Sensor Mammalian Target of Rapamycin (mTOR). Front. Immunol..

[30] van der Hee B., Wells J.M. (2021). Microbial Regulation of Host Physiology by Short-chain Fatty Acids. Trends Microbiol.

[31] Peng Q., Alqahtani S., Nasrullah M.Z.A., Shen J. (2021). Functional evidence for biased inhibition of G protein signaling by YM-254890 in human coronary artery endothelial cells. Eur J Pharmacol.

[32] Taniguchi M. (2003). YM-254890, a Novel Platelet Aggregation Inhibitor Produced by Chromobacterium sp. QS3666. J. Antibiot..

[33] Gregory R.I. (2001). DNA Methylation Is Linked to Deacetylation of Histone H3, but Not H4, on the Imprinted Genes Snrpnand U2af1-rs1. Mol. Cell. Biol..

[34] Titus L.S., A. S. C., Sharma D., Kim M.S., D’Mello S.R. (2019). The Bdnf and Npas4 genes are targets of HDAC3-mediated transcriptional repression. BMC Neurosci.

[35] Leus N.G.J. (2016). HDAC 3-selective inhibitor RGFP966 demonstrates anti-inflammatory properties in RAW 264.7 macrophages and mouse precision-cut lung slices by attenuating NF-κB p65 transcriptional activity. Biochem Pharmacol.

[36] Helft J. (2015). GM-CSF Mouse Bone Marrow Cultures Comprise a Heterogeneous Population of CD11c+MHCII+ Macrophages and Dendritic Cells. Immunity.

[37] Moran T.P., Nakano H., Kondilis-Mangum H.D., Wade P.A., Cook D.N. (2014). Epigenetic Control of *Ccr7* Expression in Distinct Lineages of Lung Dendritic Cells. J.I..

[38] Na J., Gu, Seok (2016). GM-CSF Grown Bone Marrow Derived Cells Are Composed of Phenotypically Different Dendritic Cells and Macrophages. Mol Cells.

[39] Kirkling M.E. (2018). Notch Signaling Facilitates In Vitro Generation of Cross-Presenting Classical Dendritic Cells. Cell Rep.

[40] Cyran L. (2022). Flt3L, LIF, and IL-10 combination promotes the selective in vitro development of ESAM ^low^ cDC2B from murine bone marrow. Eur J Immunol.

[41] Bolognini D., Tobin A.B., Milligan G., Moss C.E. (2016). The Pharmacology and Function of Receptors for Short-Chain Fatty Acids. Mol Pharmacol.

[42] Chriett S. (2019). Prominent action of butyrate over β-hydroxybutyrate as histone deacetylase inhibitor, transcriptional modulator and anti-inflammatory molecule. Sci Rep.

[43] Lawless S.J. (2017). Glucose represses dendritic cell-induced T cell responses. Nat Commun.

[44] Qian T. (2019). Regulation of CD11b by HIF-1α and the STAT3 signaling pathway contributes to the immunosuppressive function of B cells in inflammatory bowel disease. Mol Immunol.

[45] Zhao Y. (2018). mTOR masters monocyte development in bone marrow by decreasing the inhibition of STAT5 on IRF8. Blood.

[46] Gupta M., Han J.J., Stenson M., Wellik L., Witzig T.E. (2012). Regulation of STAT3 by histone deacetylase-3 in diffuse large B-cell lymphoma: implications for therapy. Leukemia.

[47] Lin Q. (2015). Epigenetic program and transcription factor circuitry of dendritic cell development. Nucleic Acids Res.

[48] Chrisikos T.T. (2020). STAT3 Inhibits CD103+ cDC1 Vaccine Efficacy in Murine Breast Cancer. Cancers.

[49] Li H.S. (2016). Bypassing STAT3-mediated inhibition of the transcriptional regulator ID2 improves the antitumor efficacy of dendritic cells. Sci. Signal..

[50] Hasegawa H., Matsumoto T. (2018). Mechanisms of Tolerance Induction by Dendritic Cells In Vivo. Front. Immunol..

[51] Canani R.B. (2011). Potential beneficial effects of butyrate in intestinal and extraintestinal diseases. WJG.

[52] Nastasi C. (2015). The effect of short-chain fatty acids on human monocyte-derived dendritic cells. Sci Rep.

[53] Nastasi C. (2017). Butyrate and propionate inhibit antigen-specific CD8+ T cell activation by suppressing IL-12 production by antigen-presenting cells. Sci Rep.

[54] Svolos V. (2019). Treatment of Active Crohn’s Disease With an Ordinary Food-based Diet That Replicates Exclusive Enteral Nutrition. Gastroenterology.

