# Supplementary material for: Microbiota-derived butyrate inhibits cDC development via HDAC inhibition, diminishing their ability to prime T cells
Source: Mucosal Immunol. 2024 Dec;17(6):1199–211. doi: 10.1016/j.mucimm.2024.08.003 (PMC11631772; doi:10.1016/j.mucimm.2024.08.003)

Supplementary figures:

Figure S1. Colonic cDC niche is altered in GF animals compared to SPF. A) Heatmap of top 10 differentially expressed genes within the ten cDC clusters isolated from the single cells RNA sequencing dataset previously published by Kang *et al.(*2019) B) tSNE plot of GF and SPF cDC coloured by DC1(teal) or DC2 lineage(corral). C) Percentage of GF(blue) or SPF(yellow) cells in total cDC1 or cDC2 clusters from merged dataset. D) tSNE plot of GF and SPF cDC coloured by clusters identified by DEGs. E) Percentage of GF(blue) or SPF(yellow) cells in 10 clusters from merged dataset.

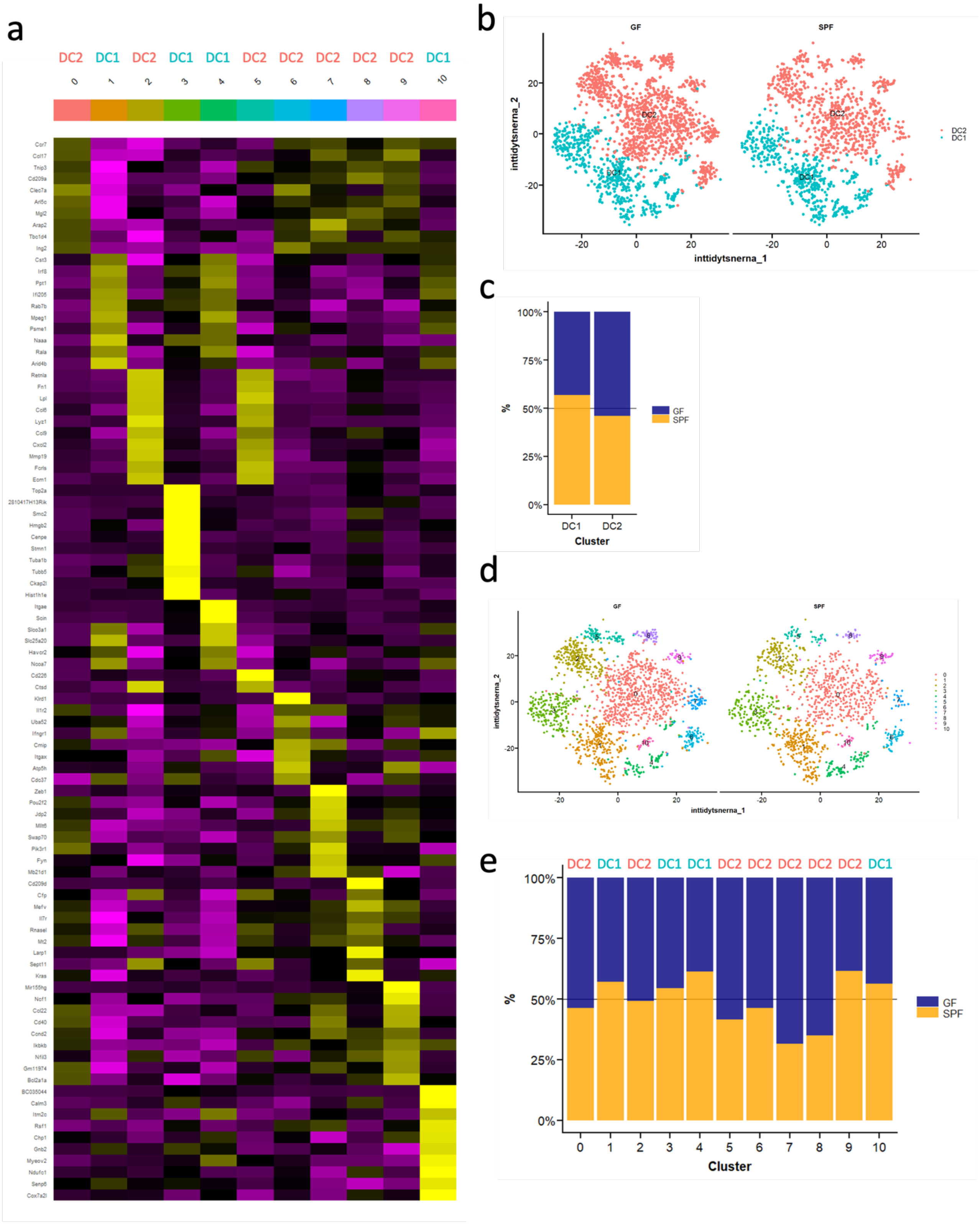
a
DC2 DC1 DC2 DC1 DC1 DC2 DC2 DC2 DC2 DC2 DC1
b
GF
SPF
inttidytsnerna_2
inttidytsnerna_1
DC2
DC1
c
100%
75%
50%
25%
0%
%
GF
SPF
DC1 DC2
Cluster
d
GF
SPF
inttidytsnerna_2
inttidytsnerna_1
e
DC2 DC1 DC2 DC1 DC1 DC2 DC2 DC2 DC2 DC2 DC1
100%
75%
50%
25%
0%
%
GF
SPF
0 1 2 3 4 5 6 7 8 9 10
Cluster

Figure S2. Gating strategy for the identification of intestinal cDC subsets. Conventional DC were selected as live single CD45+ CD3- B220- CD64- MHCII+ CD11c+ cells and then further divided based on their CD103 and CD11b expression. The data are representative of more than 15 independent experiments.

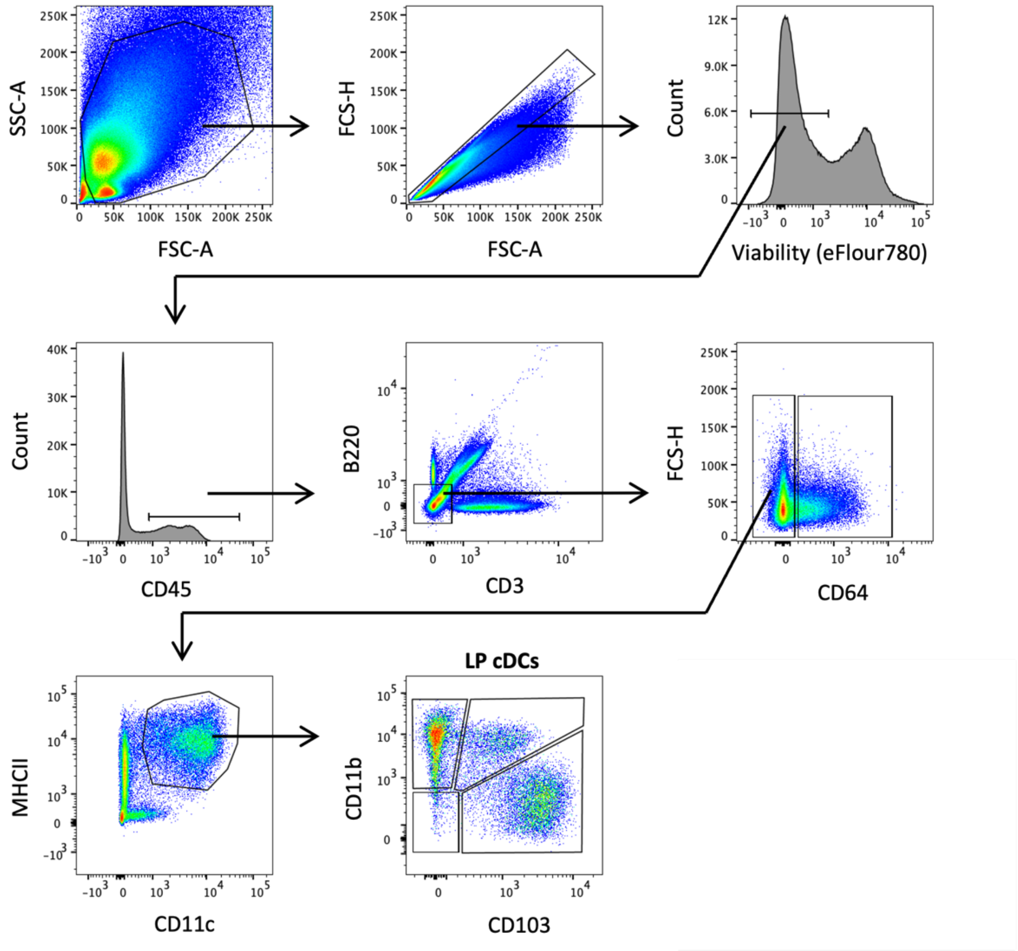

Figure S3. **Cellular changes upon butyrate administration** A) Quantification of overall cellularity in the colon in control mice, in mice treated with antibiotic cocktail (ABX) and in mice treated with antibiotics and supplemented with 200mM butyrate (ABX + Butyrate). Cells were identified as live, single, CD45+, CD3-, B220- cells. Data shown are pooled from 3 independent experiments (n=4 in each). B) Breakdown of individual experiments to analyse colonic cDC populations in presence of altered abundance of SCFA. Frequencies amongst total cDC (top panel) and absolute numbers (bottom panel) of colonic LP cDC subsets in control mice, in mice treated with antibiotic cocktail (ABX) and in mice treated with antibiotics and supplemented with 200mM butyrate (ABX + Butyrate) in 6 individual experiments (I-VI). C) CD86 expression quantified as a fold change of MFI. DN = CD11b$^-$ CD103$^-$ double negative, single CD11b$^+$ = CD11b$^+$CD103$^-$, DP = CD11b$^+$CD103$^+$ double positive and single CD11b$^-$CD103$^+$ (CD103$^+$). The data shown are fromA) pooled 3 independent experiments(n=4 each), B) 6 independent experiments with n=3-5 in each C) pooled 3 independent experiments (n=3-5 each). Each data point represents one mouse with bars representing the means. *p<0.05, **p<0.01, ***p<0.001, ****p<0.0001 as assessed by Student's t-test **(A, B),** one-way ANOVA (**D**) or two-way ANOVA with Šídák's post-test correction for multiple comparisons (**F, G**). ns=not significant

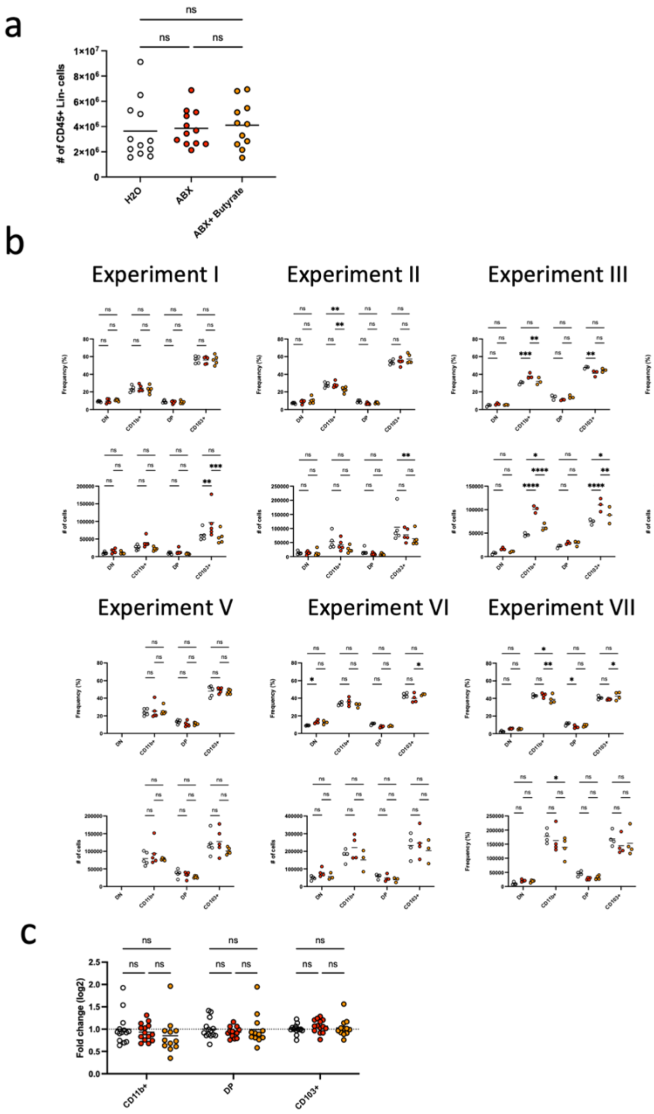

Figure S4. Effects of butyrate on antigen presenting activity of colonic cDC *in vivo* after 14 day supplementation.

A) Fold change of proportions of CTV$^-$ of OT-II CD4$^+$ T cells after 3 days of co-culture with OVA-pulsed colonic cDC populations calculated based on control group. **E-G)** Fold change of proportions of naïve OTII CD4$^+$ T cells expressing CD44 (B), CD69 (C) and Foxp3 (D) after 3 days of co-culture with OVA-pulsed colonic cDC populations calculated based on control group. Cells pre-gated on live, single, CD45$^+$, B220$^-$, CD3$^-$, CD64$^-$, CD11c$^+$, MHC$^+$ cells(A-B). All T cells pre-gated on live, single, CD3$^+$, CD4$^+$ cells (C-G). Data shown are from 1 experiment with n=3-5 *p<0.05, **p<0.01, ***p<0.001,****p<0.0001 as assessed by two-way ANOVA with Šídák's post-test correction for multiple comparisons. ns=not significant.

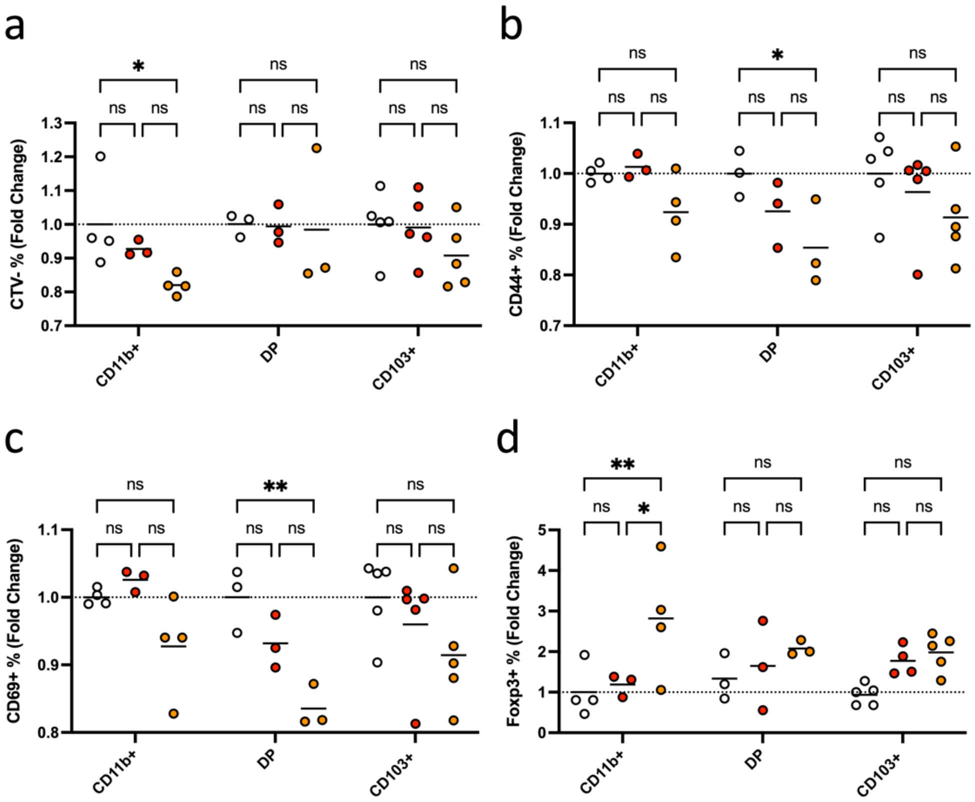

Figure S5. Gating strategy for isolating BM pre-cDC.
A) Pre-cDC were selected as live single CD3- CD19- NK1.1- CD11b- MHCII- CD11c+ B220- CCR9- CD135+ cells. Gates were based on appropriate isotype controls and unstained cells.
B) Acquisition of sorted cells yielded >95% purity in all individual gates.

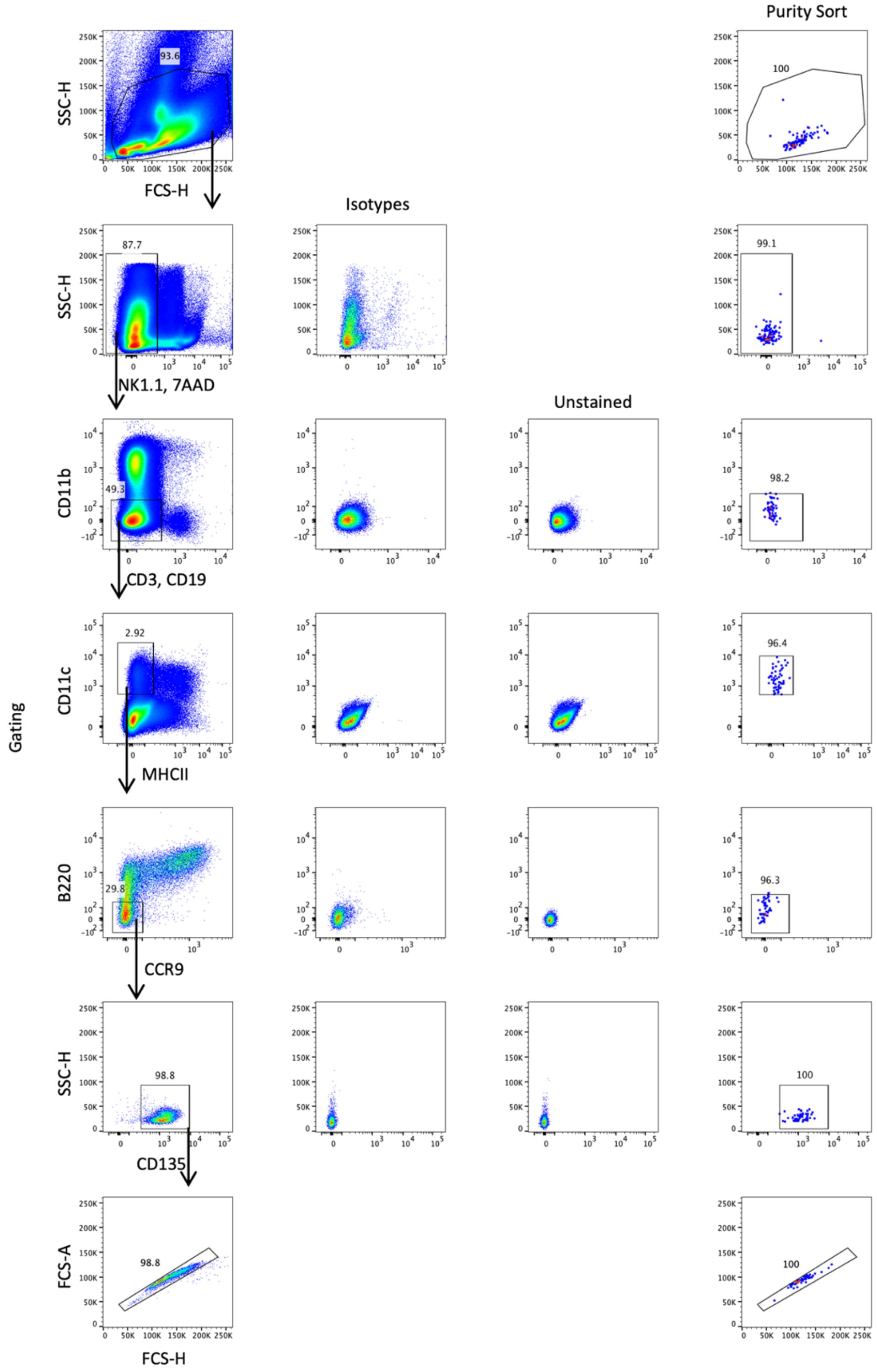

Figure S6. Identification of intestine-like cDC from pre-cDC *in vitro.*
A) Flow cytometrically sorted pre-cDC were cultured for 4 days in complete medium containing 400ng/mL Flt3 ligand and 20ng/mL GM-CSF. Live, single CD45+ cells were assessed for expression of CD64, Siglec-H, Ly6C, MHCII, CD11c, CD11b and CD103. Data are representative of at least 5 independent experiments.

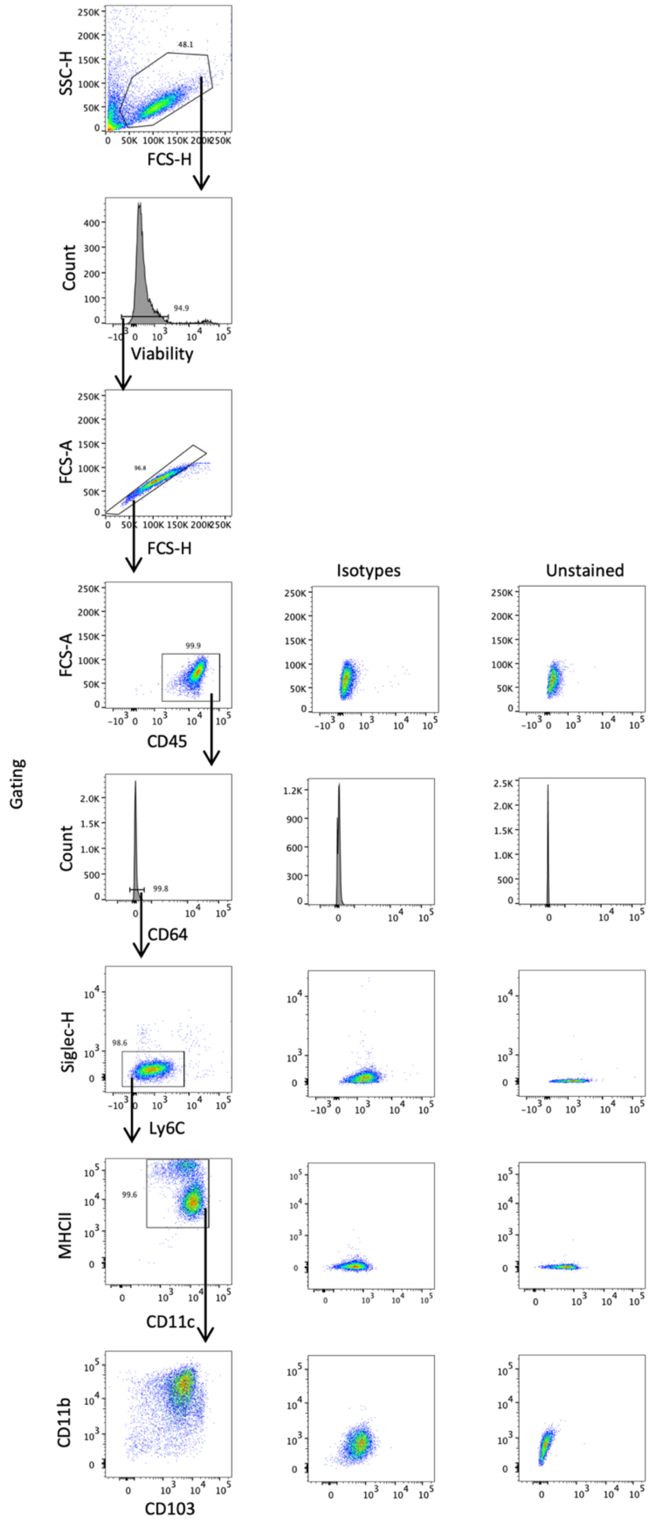

Figure S7. Validation of DC lineage markers in cells generated from pre-cDC *in vitro.* Flow cytometrically sorted pre-cDC were cultured for 4 days in complete medium containing 400ng/mL Flt3 ligand and 20ng/mL GM-CSF. Live, single CD45^{+}MHCI^{+}CD11c^{+} cells were assessed for expression of CD11b, CD103, SIRPα, CLEC9A, IRF4, IRF8, CD101. A) CLEC9A expression illustrated as heatmap dotplot (left) or as histograms (right) in CD11b$^{+/hi}$ and CD103^{hi} cell populations. B) IRF8 expression illustrated as heatmap dotplot (left) or as histograms (right) in CD11b$^{+/hi}$ and CD103^{hi} cell populations. C) SIRPα expression illustrated as heatmap dotplot (left) or as histograms (right) in CD11b$^{+/hi}$ and CD103^{hi} cell populations. D) IRF4 expression illustrated as heatmap dotplot (left) or as histograms (right) in CD11b$^{+/hi}$ and CD103^{hi} cell populations. E) CD103 expression illustrated as heatmap dotplot (left) or as histograms (right) in SIRP$\alpha^{+/hi}$ and CLEC9A^{hi} cell populations. F) IRF8 expression illustrated as heatmap dotplot (left) or as histograms (right) in SIRP$\alpha^{+/hi}$ and CLEC9A^{hi} cell populations. G) CD11b expression illustrated as heatmap dotplot (left) or as histograms (right) in SIRP$\alpha^{+/hi}$ and CLEC9A^{hi} cell populations. H) IRF4 expression illustrated as heatmap dotplot (left) or as histograms (right) in SIRP$\alpha^{+/hi}$ and CLEC9A^{hi} cell populations. I) SIRPα expression illustrated as heatmap dotplot (left) or as histograms (right) in IRF4^{+} and IRF8^{+} cell populations. J) CD103 expression illustrated as heatmap dotplot (left) or as histograms (right) in IRF4^{+} and IRF8^{+} cell populations. K) CD11b expression illustrated as heatmap dotplot (left) or as histograms (right) in IRF4^{+} and IRF8^{+} cell populations. L) CLEC9A expression illustrated as heatmap dotplot (left) or as histograms (right) in IRF4^{+} and IRF8^{+} cell populations. M) CD101 expression illustrated as histograms (right) in CD11b$^{+/hi}$ and CD103^{hi} cell populations (left). N) Histogram and dot plots illustrating expression of XCR1(MFI), gated on live CD45+, CD64-, Ly6C-, CD11c+, MHCII+ cells. Bars represent means. Data shown are from 1 experiment, representative of 2-3 independent experiments (n=3 each). Two-tailed, unpaired Student's *t*-test was applied for statistical analysis.

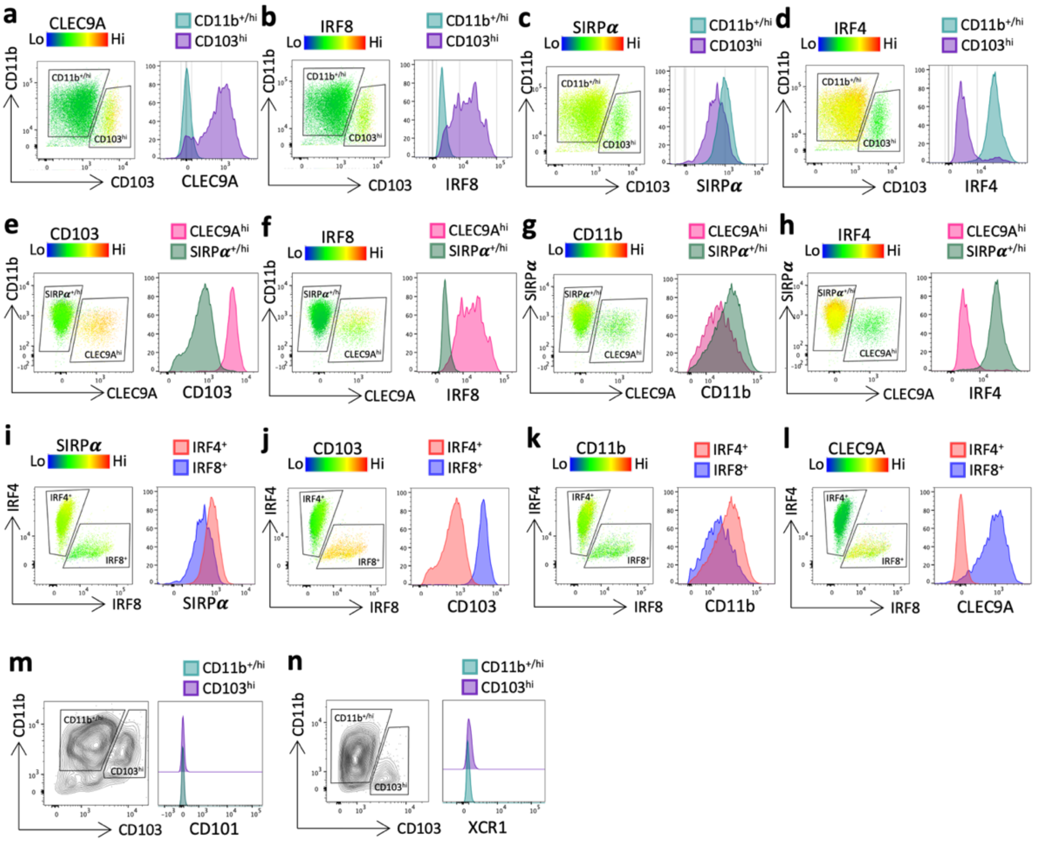

Figure S8. **Dose dependant effects of butyrate on *in vitro* generated cDC.** Dose dependent effects of butyrate on pre-cDC differentiation *in vitro.* FACS isolated pre-cDC were cultured for 4 days in complete medium containing 400ng/mL Flt3 ligand and 20ng/mL GM-CSF, in the presence or absence of 0.0625mM – 1mM butyrate. A) Histograms showing the proportion of live cells, assessed by eFluor780 dye staining. **B**) Proportion (%) and C) numbers of viable cells obtained after culture. D) Histograms showing the proportion of live cells within either DC1-like cells (Gated $CD45^+$, $CD11c^+$, $MHCII^+$, $CD103^{hi}$) (left panel) or DC2-like cells (Gated $CD45^+$, $CD11c^+$, $MHCII^+$, $CD11b^{+/hi}$) as assessed by using eFluor780 dye staining. E) Proportion (%) of viable cells within DC1-like cells. F) Proportion (%) of viable cells within DC2-like cells. G) Contour plot of *in vitro* cultured pre-cDC in the presence or absence of 0.0625mM – 1mM butyrate. Gating based on CD11b and CD103 as DC1-like cells (Gated $CD45^+$, $CD11c^+$, $MHCII^+$, $CD103^{hi}$) (left panel) or DC2-like cells (Gated $CD45^+$, $CD11c^+$, $MHCII^+$, $CD11b^{+/hi}$). H) Proportion of DC1-like cells and DC2-like cells *in vitro* cultured pre-cDC in the presence or absence of 0.0625mM – 1mM butyrate. I) Absolute numbers retrieved of DC1-like cells and DC2-like cells after *in vitro* culture of pre-cDC in the presence or absence of 0.0625mM – 1mM butyrate. Data shown are from 1 experiment (A-F) or two pooled experiments (G-I). Bars representing the means. *$p<0.05$, **$p<0.01$, ***$p<0.001$, ****$p<0.0001$ as assessed by one-way ANOVA with a follow-up Dunnett's test for multiple comparisons comparing control group with the 0.0625mM – 1mM butyrate groups(B, C, E, F) or by two-way ANOVA with Šídák's post-test correction for multiple comparisons (H, I), ns=not significant.

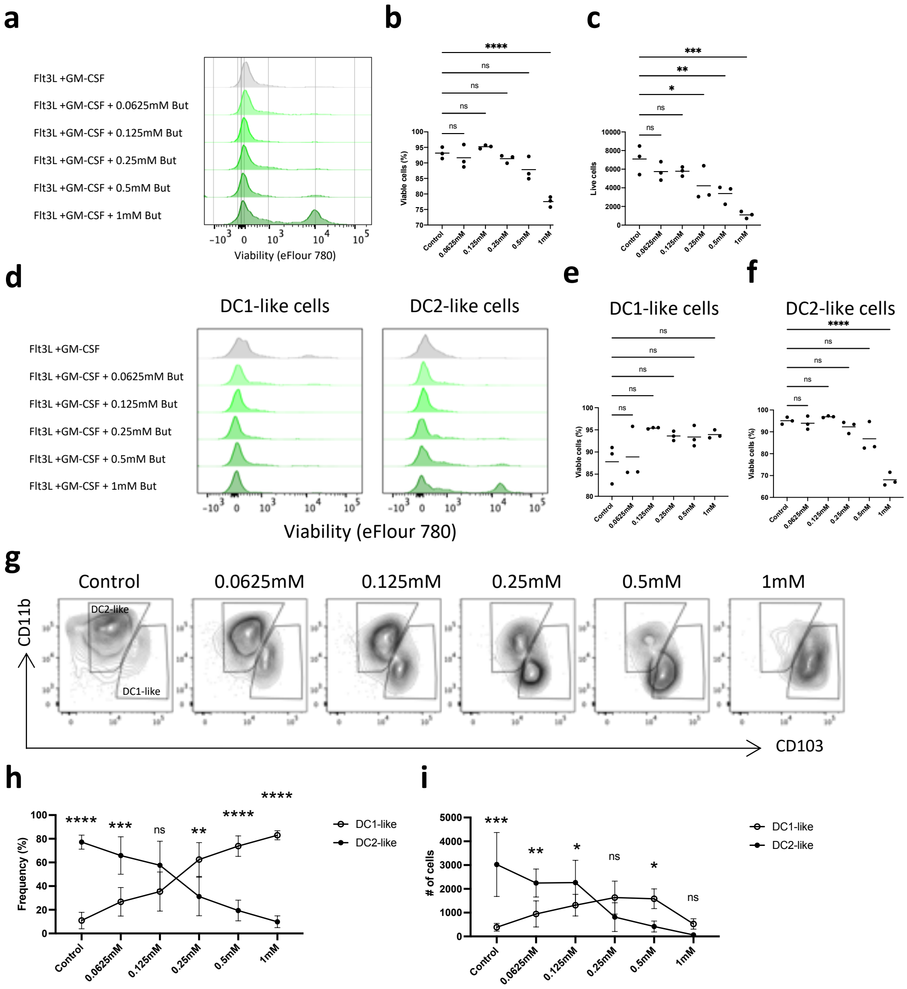
a
Flt3L +GM-CSF
Flt3L +GM-CSF + 0.0625mM But
Flt3L +GM-CSF + 0.125mM But
Flt3L +GM-CSF + 0.25mM But
Flt3L +GM-CSF + 0.5mM But
Flt3L +GM-CSF + 1mM But
Viability (eFlour 780)
b
Viable cells (%)
Control
0.0625mM
0.125mM
0.25mM
0.5mM
1mM
c
Live cells
d
DC1-like cells
DC2-like cells
Viability (eFlour 780)
e
DC1-like cells
f
DC2-like cells
g
Control
0.0625mM
0.125mM
0.25mM
0.5mM
1mM
DC2-like
DC1-like
CD11b
CD103
h
Frequency (%)
DC1-like
DC2-like
i
# of cells

Figure S9. **Optimisation of butyrate effects on *in vitro* generated cDC.** Flow cytometrically sorted pre-cDC were cultured for 4 days in complete medium containing 400ng/mL Flt3 ligand and 20ng/mL GM-CSF or in the presence or absence of 0.5mM butyrate. Live, single CD45+ cells were assessed for expression of CD64, Ly6C, MHCII, CD11c, Data are representative of at least 2 independent experiments.

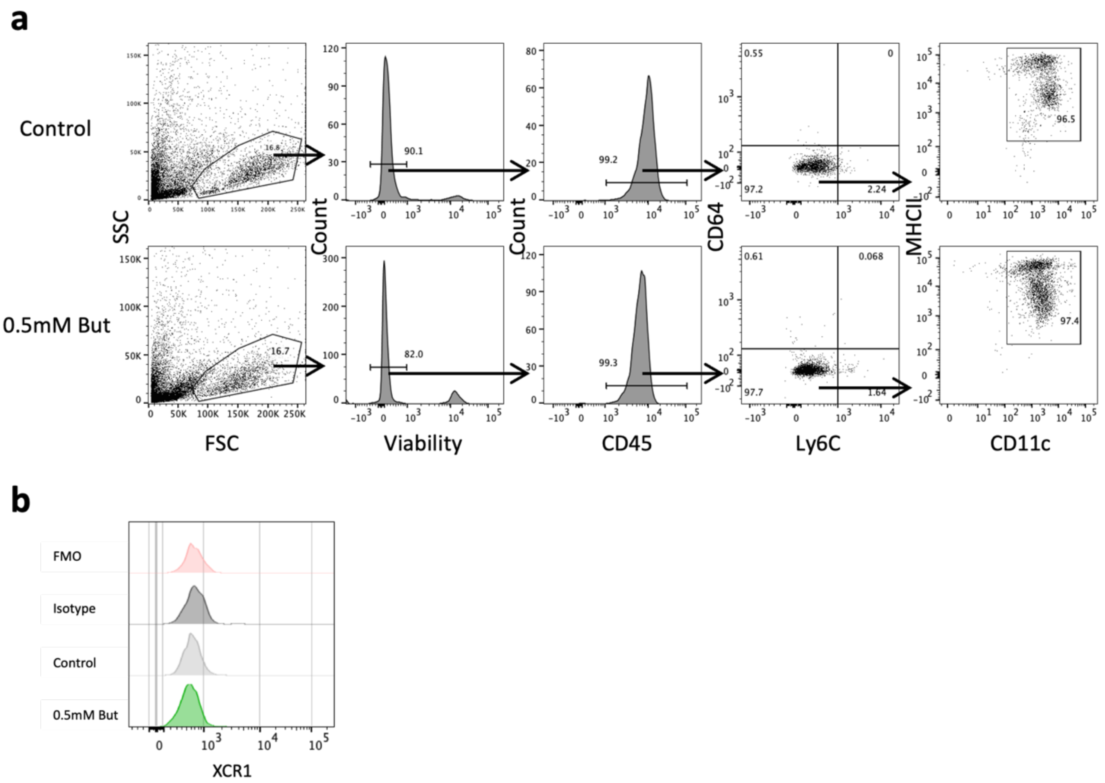

Figure S10. Ki67, pS6, H3K25Ac and H3K27Me3 levels in colonic cDC populations

A) Fold change of $Ki67^+$ proportion of cells in colonic LP cDC in control mice, in mice treated with antibiotic cocktail (ABX) and in mice treated with antibiotics and supplemented with 200mM butyrate (ABX + Butyrate). B) Fold change of pS6 (MFI) in colonic LP cDC in control mice, in mice treated with antibiotic cocktail (ABX) and in mice treated with antibiotics and supplemented with 200mM butyrate (ABX + Butyrate). C) Fold change of H3K25Ac(MFI) in colonic LP cDC in control mice, in mice treated with antibiotic cocktail (ABX) and in mice treated with antibiotics and supplemented with 200mM butyrate (ABX + Butyrate). D) Fold change of H3K25Me3(MFI) in colonic LP cDC in control mice, in mice treated with antibiotic cocktail (ABX) and in mice treated with antibiotics and supplemented with 200mM butyrate (ABX + Butyrate). The data shown are pooled from (A-C) 2 independent experiments (n=4 each) or 1 experiment (D). Fold changes were calculated based on cotrol group values. Single $CD11b^+$ = $CD11b^+CD103^-$, DP = $CD11b^+CD103^+$ double positive and single $CD11b^-CD103^+$ ($CD103^+$). *p<0.05, **p<0.01, ***p<0.001,as assessed by two-way ANOVA with Šídák's post-test correction for multiple comparisons, ns=not significant.

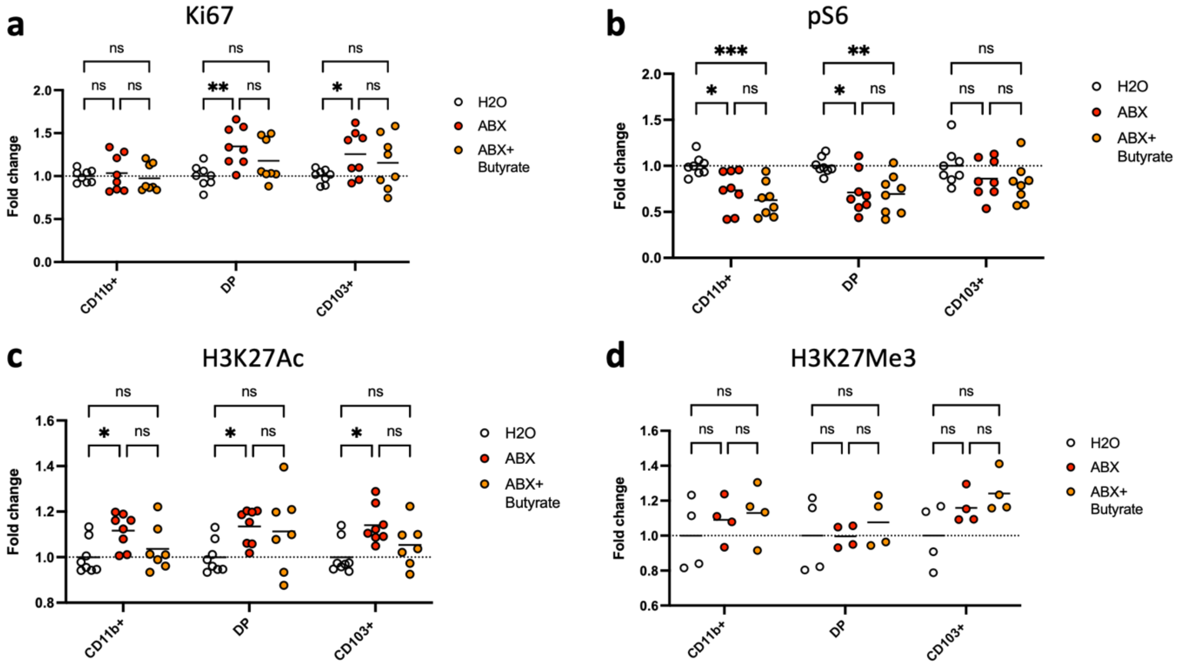

Supplement: Supplementary Data 1 [file mmc1.pdf]
